# Engineering of *Helicobacter pylori* Dimeric Oxidoreductase DsbK (HP0231)

**DOI:** 10.3389/fmicb.2016.01158

**Published:** 2016-07-26

**Authors:** Katarzyna M. Bocian-Ostrzycka, Magdalena J. Grzeszczuk, Anna M. Banaś, Katarzyna Jastrząb, Karolina Pisarczyk, Anna Kolarzyk, Anna M. Łasica, Jean-François Collet, Elżbieta K. Jagusztyn-Krynicka

**Affiliations:** ^1^Department of Bacterial Genetics, Faculty of Biology, Institute of Microbiology, University of WarsawWarsaw, Poland; ^2^Walloon Excellence in Life Sciences and BiotechnologyBrussels, Belgium; ^3^de Duve Institute, Université Catholique de LouvainBrussels, Belgium

**Keywords:** *Helicobacter pylori*, disulfide bonds, Dsb proteins, oxidoreductases, chaperone activity, site-directed mutagenesis, protein engineering

## Abstract

The formation of disulfide bonds that are catalyzed by proteins of the Dsb (*disulfide bond*) family is crucial for the correct folding of many extracytoplasmic proteins. Thus, this formation plays an essential, pivotal role in the assembly of many virulence factors. The *Helicobacter pylori* disulfide bond-forming system is uncomplicated compared to the best-characterized *Escherichia coli* Dsb pathways. It possesses only two extracytoplasmic Dsb proteins named HP0377 and HP0231. As previously shown, HP0377 is a reductase involved in the process of cytochrome c maturation. Additionally, it also possesses disulfide isomerase activity. HP0231 was the first periplasmic dimeric oxidoreductase involved in disulfide generation to be described. Although HP0231 function is critical for oxidative protein folding, its structure resembles that of dimeric EcDsbG, which does not confer this activity. However, the HP0231 catalytic motifs (CXXC and the so-called *cis*-Pro loop) are identical to that of monomeric EcDsbA. To understand the functioning of HP0231, we decided to study the relations between its sequence, structure and activity through an extensive analysis of various HP0231 point mutants, using *in vivo* and *in vitro* strategies. Our work shows the crucial role of the *cis*-Pro loop, as changing valine to threonine in this motif completely abolishes the protein function *in vivo*. Functioning of HP0231 is conditioned by the combination of CXXC and the *cis*-Pro loop, as replacing the HP0231 CXXC motif by the motif from EcDsbG or EcDsbC results in bifunctional protein, at least in *E. coli*. We also showed that the dimerization domain of HP0231 ensures contact with its substrates. Moreover, the activity of this oxidase is independent on the structure of the catalytic domain. Finally, we showed that HP0231 chaperone activity is independent of its redox function.

## Introduction

The ability of proteins to fold into their correct three-dimensional structure is vital for cell growth and survival. Cysteine—an amino acid containing a thiol (-SH) group—often plays a crucial role in protein folding. The oxidation reaction between thiol groups of two cysteine residues results in the formation of a disulfide bond. Disulfide bond formation is a rate-limiting step in the protein-folding process, and it is catalyzed by oxidoreductases. In Gram-negative bacteria, disulfide bond formation takes place in the oxidative environment of the periplasm, where it is catalyzed by a set of soluble and membrane-bound Dsb (disulfide bond) proteins. This process is critical for the correct folding and structural stability of many secreted and membrane proteins. Therefore, it plays an essential role in the assembly of many virulence factors (Godlewska et al., 2006; Lasica and Jagusztyn-Krynicka, 2007; Heras et al., 2009; Bocian-Ostrzycka et al., 2015a). Two periplasmic proteins are of great importance for disulfide formation: DsbA and DsbC. The mechanism of their action has been studied in great detail for *Escherichia coli* (Shouldice et al., 2011; Denoncin and Collet, 2013). EcDsbA is the main periplasmic oxidase involved in disulfide bond formation. However, it acts in a non-selective way, introducing disulfides between consecutive cysteine residues that are present in the polypeptide chain just after it enters the periplasm, or even during its transfer throughout the inner membrane. So, in the case of proteins whose correct folding requires the presence of disulfide bonds between non-consecutive cysteine residues, DsbA activity results in their improper, mis-oxidized conformation. The isomerase DsbC is responsible for reshuffling these incorrectly introduced disulfides (Berkmen, 2012; Cho and Collet, 2013). DsbC is kept in the reduced form by an integral membrane protein, DsbD, that catalyzes the transfer of electrons from the cytoplasm to the periplasm. Another membrane protein, DsbB, provides disulfides to DsbA by generating them from quinone reduction (Inaba and Ito, 2008; Cho and Collet, 2013).

DsbA, the first Dsb protein discovered, has attracted the most scientific interest. From the first description of EcDsbA in 1991 by Bardwell et al., numerous studies using a combination of biochemical, genetic and structural methods have characterized EcDsbA in great detail (Bardwell et al., 1991; Shouldice et al., 2011; Berkmen, 2012). Recently, many homologs of EcDsbA from other bacterial species, both Gram-negative and Gram-positive, have also been analyzed, using *in vivo* and *in vitro* strategies (Heras et al., 2009; Hatahet et al., 2014). Although these homologs share some common properties, such as a monomeric structure and a thioredoxin fold that has two essential catalytic motifs (CXXC and *cis*-Pro), they differ significantly in their structures and biochemical properties (McMahon et al., 2014).

Our understanding of dimeric Dsb oxidoreductases is less extensive than our knowledge about the monomeric homologs of EcDsbA. Most of periplasmic dimeric oxidoreductases exhibit disulfide isomerase activity, as they are involved in proofreading and repairing/shuffling of incorrectly generated disulfide bonds in proteins containing multiple cysteine residues (Rietsch et al., 1996). A second group of periplasmic dimeric oxidoreductases, on the other hand, protect single cysteine residues from oxidation to sulfenic acid (Shao et al., 2000; Depuydt et al., 2009). The intensely studied member of the first group is EcDsbC, and the best-characterized prototypical representative of the second group is EcDsbG. Both EcDsbC and EcDsbG are homodimers possessing a V-shaped structure that is composed of an N-terminal dimerization domain connected to a C-terminal TRX-fold catalytic domain by a linker region (Haebel et al., 2002; Heras et al., 2004). Although the structures of EcDsbC and EcDsbG are architecturally similar, they differ in several properties that determine the differences in their functioning, such as the size of the substrate binding cleft and the surface charge (McCarthy et al., 2000; Heras et al., 2004).

Some (though not many) bacteria possess dimeric, periplasm-located oxidoreductases involved in disulfide generation, similar to monomeric DsbA. So far, these forms of Dsb proteins have been identified in *Legionella pneumophila, Francisella tulariensis, Corynebacterium glutamicum*, and *Helicobacter pylori* (Daniels et al., 2010; Jameson-Lee et al., 2011; Qin et al., 2011, 2014). Besides their oxidative function, conditioned by a thioredoxin fold with a CXXC motif, all the above-mentioned proteins differ significantly in many details of their structures and phylogenetic origins (Schmidt et al., 2013; Bocian-Ostrzycka et al., 2015b; Lester et al., 2015). It has been shown that at least two of them, LpDsbA2 and FtDsbA, are bifunctional proteins that are simultaneously active in both the oxidizing and isomerization pathways (Qin et al., 2014; Kpadeh et al., 2015).

In the present study, we report the functional and biochemical characterization of HP0231 and its mutated variants. The *H. pylori* disulfide bond-forming system is rather simple. This bacterium does not encode classical DsbA/DsbB, nor DsbC/DsbD. It possesses only two extracytoplasmic Dsb proteins, HP0231 and HP0377. We have previously shown that HP0377 is a reductase involved in the process of cytochrome c maturation, and it also possesses disulfide isomerase activity *in vitro*. In *H. pylori* cells HP0377 is present in a reduced form and the absence of the main periplasmic oxidase HP0231 influences its redox state. Taking into account that there is no classical DsbC protein in the *H. pylori* proteome, it is highly probable that HP0377 is *in vivo* a multifunctional protein, in contrast to most CcmGs that are involved only in the cytochrome c biogenesis process (Roszczenko et al., 2015).

The subject of this study, HP0231, was previously described by our research group as a major dimeric oxidoreductase of *H. pylori* that catalyzes disulfide bond formation in the periplasm (Roszczenko et al., 2012). Lack of HP0231 affects *H. pylori* resistance to oxidative stress (Lester et al., 2015). Additionally, HP0231 activity ensures correct functioning of some virulence factors related to bacterial gastric pathology (Zhong et al., 2016). HP0231 structure has been solved and it resembles that of dimeric EcDsbG (Yoon et al., 2011). Although the solved structure of the HP0231 catalytic domain is similar in structure to class II DsbA proteins, it contains CXXC and *cis*-Pro motifs characteristic of class I DsbA proteins (McMahon et al., 2014). HP0231 is crucial for oxidative protein folding like EcDsbA, and at the same time, exhibits high chaperone activity similar to EcDsbC or EcDsbG. It lacks isomerization activity and interacts with HP0377 (Bocian-Ostrzycka et al., 2015b; Roszczenko et al., 2015). Thus, we asked this question: which elements of its structure determine these atypical properties? To evaluate the role of the catalytic motifs, we carried out extensive analysis of HP0231 point-mutated versions using *in vivo* and *in vitro* strategies. To differentiate the impact of the HP0231 dimerization domain and its linker on protein function, three fusion proteins were constructed and then analyzed for their biochemical properties and *in vivo* functioning.

## Materials and methods

### Bacterial strains, primers, plasmids, media, and growth conditions

Bacterial strains, plasmids and primers used in this study are listed in Table 1 and Supplementary Tables S1, S2. *Helicobacter pylori* strains (26695 and N6) were grown on Blood Agar base no. 2 (BA) plates (Merck) supplemented with 10% (v/v) horse blood and *Helicobacter* Selective Supplement-Dent (ThermoFisher Scientific), or on Mueller Hinton Broth (MH) supplemented with 10% (v/v) Fetal Bovine Serum (FBS) (Lonza), at 37°C under microaerobic conditions that were provided by Anoxomat Mark II OP (MART® Microbiology B.V) or CampyGen (ThermoFisher Scientific). For the selection of *H. pylori* N6 *hp0231::cat* complemented strains, kanamycin (25 μg ml^−1^) or/and chloramphenicol (10 μg ml^−1^) was added to the growth media. The *H. pylori* N6 *hp0231::cat* was employed for complementation experiments by HP0231 and its mutated forms. *E. coli* strains were grown at 37°C on solid or liquid Luria-Bertani (LB) medium or on M63 minimal medium (Hiniker et al., 2005). When needed, media were supplemented with antibiotics at the following concentrations: 100 μg ml^−1^ ampicillin, 30 μg ml^−1^ kanamycin and 20 μg ml^−1^ chloramphenicol. The *E. coli* strains JCB817 (*dsbA::kan*) and JCB818 (*dsbAB::kan*) (Bardwell et al., 1991), PL263 (*mdoGdsbC::kan)* (Leverrier et al., 2011) were employed for complementation experiments by HP0231 and its mutated forms.

**Table 1:** **Strains used in this study**.

**Lp**.	**Name**	**Relevant characteristics**	**Source/References**
***Helicobacter pylori*** **STRAINS**			
1	N6	*H. pylori* wild-type	Behrens et al., 2012
2	PR378	N6 *hp0231::cat*	Roszczenko et al., 2012
3	PR397	N6 *hp0231::cat*/pUWM397 (*hp0231^+^in trans*)	Roszczenko et al., 2012
4	KBO513	N6 *hp0231::cat*/pUWM513 (*hp0231*CPHS*^+^in trans*)	This study
5	KBO517	N6 *hp0231::cat*/pUWM517 (*hp0231*CPHA*^+^in trans*)	This study
6	KBO2031	N6 *hp0231::cat*/pUWM2031 (*hp0231*APHC*^+^in trans*)	This study
7	KBO2032	N6 *hp0231::cat*/pUWM2032 (*hp0231*APHA*^+^in trans*)	This study
8	KBO545	N6 *hp0231::cat*/pUWM545 (*hp0231*CPYC*^+^in trans*)	This study
9	KBO572	N6 *hp0231::cat*/pUWM572 (*hp0231*CGYC*^+^in trans*)	This study
10	KBO573	N6 *hp0231::cat*/pUWM573 (*hp0231*CPYC*∕*TcP*^+^in trans*)	This study
11	KBO580	N6 *hp0231::cat*/pUWM580 (*hp0231*CGYC*∕*TcP*^+^in trans*)	This study
12	KBO546	N6 *hp0231::cat*/pUWM546 (*hp0231*TcP*^+^in trans*)	This study
13	KBO2115	N6 *hp0231::cat*/pUWM2115 (hybrid *dimG_αK_catK^+^in trans*)	This study
14	KBO2116	N6 *hp0231::cat*/pUWM2116 (hybrid *dimG_αG_catK^+^in trans*)	This study
15	KBO2117	N6 *hp0231::cat*/pUWM2117 (hybrid *dimK_αK_catA^+^in trans*)	This study
***Escherichia coli*** **STRAINS**			
16	TG1	*supE44 hsdΔ 5 thi Δ(lac^−^ proAB) F' [traD36 proAB^+^lacI*q* lacZΔM15]*	Sambrook and Russel, 2001
17	BL21 (DE3)	F−*ompT hsdS*B*(r*B*^−^m*B*^−^) gal dcm lon*	Novagen
18	BL21/*EcdsbA^+^*	BL21 carrying pET28a/*EcdsbA*	JFC Collection
19	BL21/*EcdsbC^+^*	BL21 carrying pET28a/*EcdsbC*	JFC Collection
20	BL21/*EcdsbG^+^*	BL21 carrying pET28a/*EcdsbG*	JFC Collection
21	Rosetta (DE3)pLacI	F−*ompThsdS*B* (r*B*- m*B*-) gal dcm*pLacIRARE (Cmr)	Novagen
22	KBO2044	Rosetta carrying pUWM525 (*hp0231* in pET28a)	Bocian-Ostrzycka et al., 2015b
23	KBO2068	Rosetta carrying pUWM2062 (*hp0231*CPHS**in pET28a)	This study
24	KBO2067	Rosetta carrying pUWM2061 (*hp0231*CPHA**in pET28a)	This study
25	KBO2104	Rosetta carrying pUWM2103 (*hp0231*CPYC**in pET28a)	This study
26	KBO2085	Rosetta carrying pUWM2084 (*hp0231*CGYC**in pET28a)	This study
27	KBO2042	Rosetta carrying pUWM2039 (*hp0231*CPYC*∕*TcP**in pET28a)	This study
28	KBO2041	Rosetta carrying pUWM2038 (*hp0231*CGYC*∕*TcP**in pET28a)	This study
29	KBO2043	Rosetta carrying pUWM2040 (*hp0231*TcP**in pET28a)	This study
30	JCB816	MC1000 *phoR λ102*	Bardwell et al., 1991
31	JCB817	JCB 816 *dsbA::kan1*	Bardwell et al., 1991
32	JCB818	JCB 816 *dsbB::kan2*	Bardwell et al., 1991
33	KBO519	JCB816 carrying pHel2	Bocian-Ostrzycka et al., 2015b
34	PR501	JCB817 carrying pHel2	Roszczenko et al., 2012
35	PR503	JCB817 carrying pUWM500 (*HP0231^+^ in trans)*	Roszczenko et al., 2012
36	PR522	JCB818 carrying pUWM500 (*HP0231^+^ in trans)*	Roszczenko et al., 2012
37	KBO533	JCB817 carrying pUWM531 (*hp0231*CPHS*^+^in trans*)	This study
38	KBO532	JCB817 carrying pUWM530 (*hp0231*CPHA*^+^in trans*)	This study
39	KBO2059	JCB817 carrying pUWM2058 (*hp0231*APHC*^+^in trans*)	This study
40	KBO2063	JCB817 carrying pUWM2060 (*hp0231*APHA*^+^in trans*)	This study
41	KBO563	JCB817 carrying pUWM560 (*hp0231*CPYC*^+^in trans*)	This study
42	KBO565	JCB817 carrying pUWM558 (*hp0231*CGYC*^+^in trans*)	This study
43	KBO566	JCB817 carrying pUWM559 (*hp0231*CPYC*∕*TcP*^+^ in trans*)	This study
44	KBO581	JCB817 carrying pUWM579(*hp0231*CGYC*∕*TcP*^+^ in trans*)	This study
45	KBO564	JCB817 carrying pUWM557 (*hp0231*TcP*^+^in trans*)	This study
46	KBO2133	JCB817 carrying pUWM2130 (hybrid *dimG_αK_catK^+^ in trans*)	This study
47	KBO2134	JCB817 carrying pUWM2131 (hybrid *dimG_αG_catK^+^ in trans*)	This study
48	KBO2135	JCB817 carrying pUWM2132 (hybrid *dimK_αK_catA^+^ in trans*)	This study
49	PR521	JCB818 carrying pHel2	Roszczenko et al., 2012
50	KBO2149	JCB818 carrying pUWM560 (*hp0231*CPYC*^+^in trans*)	This study
51	KBO2147	JCB818 carrying pUWM558 (*hp0231*CGYC*^+^in trans*)	This study
52	KBO2148	JCB818 carrying pUWM559 (*hp0231*CPYC*∕*TcP*^+^ in trans*)	This study
53	KBO2150	JCB818 carrying pUWM579(*hp0231*CGYC*∕*TcP*^+^ in trans*)	This study
54	KBO2146	JCB818 carrying pUWM557 (*hp0231*TcP*^+^in trans*)	This study
55	KBO2142	JCB818 carrying pUWM2130 (hybrid *dimG_αK_catK^+^ in trans*)	This study
56	KBO2143	JCB818 carrying pUWM2131 (hybrid *dimG_αG_catK^+^ in trans*)	This study
57	KBO2144	JCB818 carrying pUWM2132 (hybrid *dimK_αK_catA^+^ in trans*)	This study
58	PL263	*MC1000 mdoG::kan1; dsbC::kan2*	Leverrier et al., 2011
59	PL284	PL263 (*mdoGdsbC::kan*) carrying pBAD33	Leverrier et al., 2011
60	PL285	PL263 carrying JFC355 (*dsbC^+^ in trans*)	Leverrier et al., 2011
61	KBO2087	PL263 carrying pUWM500 (*HP0231^+^ in trans)*	Bocian-Ostrzycka et al., 2015b
62	KBO2111	PL263 carrying pUWM531 (*hp0231*CPHS*^+^in trans*)	This study
63	KBO2110	PL263 carrying pUWM530 (*hp0231*CPHA*^+^in trans*)	This study
64	KBO2108	PL263 carrying pUWM560 (*hp0231*CPYC*^+^in trans*)	This study
65	KBO2106	PL263 carrying pUWM558 (*hp0231*CGYC*^+^in trans*)	This study
66	KBO2107	PL263 carrying pUWM559 (*hp0231*CPYC*∕*TcP*^+^ in trans*)	This study
67	KBO2109	PL263 carrying pUWM579 (*hp0231*CGYC*∕*TcP*^+^ in trans*)	This study
68	KBO2105	PL263 carrying pUWM557 (*hp0231*TcP*^+^in trans*)	This study
69	KBO2136	PL263 carrying pUWM2130 (hybrid *dimG_αK_catK^+^ in trans*)	This study
70	KBO2137	PL263 carrying pUWM2131 (hybrid *dimG_αG_catK^+^ in trans*)	This study
71	KBO2138	PL263 carrying pUWM2132 (hybrid *dimK_αK_catA^+^ in trans*)	This study

### General DNA manipulations

Standard DNA manipulations were carried out as described in the Sambrook manual (Sambrook and Russel, 2001) or according to the manufacturer's instructions (A&A Biotechnology, ThermoFisher Scientific). Polymerase chain reactions (PCR) were performed with PrimeStar HS DNA Polymerase (Takara) under standard conditions, according to the manufacturer's instructions. Synthetic oligonucleotides synthesis and DNA sequencing were performed by Genomed S.A., Warsaw, Poland.

#### Construction of HP0231 plasmids with site directed mutations for complementation experiments

To analyze the complementation of the *hp0231*^−^ mutation in *H. pylori* N6, and to analyze the *dsbA/dsbAB* mutants in *E. coli* JCB816 (strains JCB817 and JCB818, respectively) and the *dsbC* mutant in *E. coli* MC1000 (strain PL283) by mutated forms of HP0231, several recombinant plasmids were constructed, based on shuttle *E. coli*/*H. pylori* plasmids pHel3 and pHel2. Because the *H. pylori hp0231* mutant and the *E. coli dsbA/dsbAB* mutants carry different genes responsible for antibiotic resistance, we used two different plasmids as a starting point for these experiments. However, both plasmids have the same replication system, and thus were present at a similar copy number. Site-directed mutagenesis was performed according to the QuickChange® Site-Directed Mutagenesis Kit Protocol with minor modifications. Briefly, the reactions were performed with PrimeStar HS DNA Polymerase (Takara) using pUWM389 as a template, applying appropriate primer pairs for site-directed mutations (Supplementary Table S1). The resulting plasmids were verified by sequencing. The purified plasmids carrying point mutated versions of HP0231, as well as the shuttle plasmids, were digested with XhoI/BamHI and ligated together to form appropriate plasmids (Supplementary Table S2, positions 9–26). For the complementation tests, the plasmids based on pHel3 were introduced into *H. pylori* N6 lacking *hp0231*, and the plasmids based on pHel2 were used for the *E. coli* lacking *dsbA*/*dsbAB/dsbC* (Table 1).

#### Construction of the vectors carrying *EcdsbG-hp0231* and *hp0231-EcdsbA* chimeras for complementation assays

“Hybrid” proteins designed in this study are listed in Supplementary Table S2. All genes coding hybrid proteins were cloned under the promoter of the *hp0231* gene, with its native signal sequence. Briefly, primers listed in Supplementary Table S1 (positions 3–13) were used to amplify the DNA regions encoding the promoter, signal sequence, dimerization domain, α-linker and catalytic domain regions of the *hp0231* gene from the chromosome of *H. pylori* 26695 or the *EcdsbG/EcdsbA* genes from the chromosome of *E. coli* TG1. The inner primers contained 5′ leader nucleotide sequences complementary to each other. Each PCR product was purified by a Gel-Out Concentrator extraction kit (A&A Biotechnology). Next, a mixture of the intermediate purified products (in equal amounts) was used as a template in a single PCR reaction, using the primers HP231_BamL/HP231His_XhoR3 or HP231_BamL/DsbAkat_HisXho for the *EcdsbG-hp0231* and *hp0231-EcdsbA* chimeras, respectively (Table 2 and Supplementary Table S1). Subsequently, the resulting PCR products were purified and cloned into pJET1.2 using CloneJET PCR Cloning Kit (ThermoFisher Scientific) to generate intermediate plasmids. Finally, using BamHI and XhoI restriction enzymes, the 1.3–1.5 kb DNA regions encoding hybrid proteins were transferred into pHel2 and pHel3, generating the plasmids listed in Supplementary Table S2, positions 27–32. Correct construction of the resulting plasmids was verified by sequencing. Production of the proper proteins was confirmed by Western-blot, using anti-HP0231 serum or anti-His antibodies (Ni-NTA HRP Conjugate; QIAGEN). Anti-HP0231 serum was previously produced by rabbit immunization in the Animal Facility, Faculty of Biology, University of Warsaw (Roszczenko et al., 2012).

**Table 2:** **Design of protein chimeras**.

	**Hybrid protein**	**Signal sequence**	**Dimerization domain**	**α-linker**	**Catalytic domain**	**Primer pairs (numbers in Supplementary Table S1)**			
						**Promoter region**	**Dimerization domain/α-linker**	**Catalytic domain**	**Ligation PCR**
1	dimGαKcatK	aa 1–28 HP0231	aa 20–58 EcDsbG	aa 97–130 HP0231	aa 131–265 HP0231	6,7	8,9	10,13	6,13
2	dimGαGcatK	aa 1–28 HP0231	aa 20–58 EcDsbG	aa 79–104 EcDsbG	aa 131–265 HP0231	6,7	8,11	12,13	6,13
3	dimKαKcatA	aa 1–28 HP0231	aa 29–97 HP0231	aa 97–130 HP0231	aa 20–208 EcDsbA	6,14		15,16	6,16

#### Natural transformation of *H. pylori*

The naturally competent *H. pylori* N6 *hp0231::cat* was mixed with appropriate plasmid DNA and grown on BA plates supplemented with chloramphenicol or kanamycin as previously described (Roszczenko et al., 2012; Bocian-Ostrzycka et al., 2015b).

### Protein analysis and biochemical assays

#### Overexpression and purification of proteins for biochemical experiments

All the proteins used for biochemical experiments were overexpressed from *E. coli* BL21 or Rosetta strains harboring the appropriate plasmids (Supplementary Table S2, positions 33–43) by autoinduction (Studier, 2005) or IPTG induction (Roszczenko et al., 2012). The expression vectors carrying HP0231-mutated forms (Supplementary Table S2, positions 34–40) were constructed by amplifying the region encoding the mature catalytic domain of tested proteins with primers 231expI and 231expII. For cloning the insert into pET28a and to create the HP0231mut-His_6_ recombinant proteins, NcoI and XhoI restriction enzymes were used to yield plasmids. For biochemical experiments, proteins were expressed and purified from *E. coli* Rosetta that harbored appropriate plasmids (strains listed in Table 1, positions 22–29). EcDsbA, EcDsbC or EcDsbG *E. coli* proteins were overexpressed from *E. coli* BL21 harboring pET28a/EcDsbA, EcDsbC or EcDsbG (JFC lab, Table 1, positions 18–20). All proteins were purified as described earlier (Bocian-Ostrzycka et al., 2015b).

#### Determination of the *in vivo* redox state of proteins

The redox states of HP0231-mutated forms were visualized by alkylating the free cysteine residues using 4-acetamido-4′-maleimidylstilbene-2,2′-disulfonic acid (AMS, ThermoFisher Scientific) (Roszczenko et al., 2012; Bocian-Ostrzycka et al., 2015b) or MalPEG5000 (Methoxypolyethylene glycol maleimide-5000 Da; Sigma) (Kpadeh et al., 2013). These agents can only modify covalently free thiols, resulting in a molecular mass increase of 490 Da (AMS) or 5 kDa (MalPEG). Briefly, *H. pylori* cells were harvested from BA plates after 24 or 48 h of incubation under microaerobic conditions. *E. coli* cells were incubated overnight in LB-medium in standard conditions. Samples were standardized using OD_600_ of the culture, and ice-cold trichloroacetic acid (TCA, final concentration 10% v/v) was immediately added directly to the culture. Whole-cell proteins were precipitated and collected by centrifugation, washed with ice-cold acetone, and then dissolved in 50 mM Tris-HCl (pH 7.5), 10 mM EDTA, 0.1% (v/v) SDS containing 20 mM AMS or MalPEG5000 by agitation (1400 rpm) for 60 min at 37°C. The proteins in non-reducing Laemmli buffer were resolved by 18% (for AMS) and 12% (for MalPEG5000) SDS-PAGE without reducing agent. Proteins were then detected by Western-blot analysis using a specific serum. As controls, we used samples previously treated with 100 mM DTT for 60 min at 30°C before precipitation of the proteins with TCA.

#### Alkaline phosphatase (AP) assay

The ability of HP0231-mutants to restore the activity of alkaline phosphatase *in vivo* in *E. coli* cells was determined in minimal medium M63 as previously described (Roszczenko et al., 2012; Bocian-Ostrzycka et al., 2015b).

#### Insulin reduction assay

Reductase activity was assessed by an insulin precipitation assay (Bardwell et al., 1991; Kpadeh et al., 2013) using human insulin solution (Sigma) (Bocian-Ostrzycka et al., 2015b). Reactions (triplicate) were carried out in 200 μl of 100 mM sodium phosphate buffer, pH 7.0, 133 μM insulin, 1 mM dithiothreitol (DTT), 2 mM EDTA and 10 μM of HP0231-mutated forms or EcDsbA; reaction mixtures were incubated in a 96-well plate format at room temperature in a Sunrise™ (Tecan) plate reader. Reactions were started by adding DTT to a final concentration of 1 mM. The changes in the absorbance (A_650_) as a function of time were measured (Collet et al., 2003; Kpadeh et al., 2013). The results are presented as the average of three independent experiments.

#### Chaperone activity of HP0231 mutants

The chaperone activity of HP0231 mutants, in comparison to EcDsbG and/or HP0231, was determined as described previously (Shao et al., 2000; Bocian-Ostrzycka et al., 2015b) using thermal aggregation of citrate synthase (CS, Sigma) as the chaperone substrate protein. Briefly, reactions (triplicate) were carried out in 2 ml of 40 mM HEPES, pH 7.5, 0.15 μM CS, and 0.2 μM of HP0231-mutated forms; using 0.2 μM EcDsbG or HP0231 as a positive controls and BSA as a negative control; all reactions were at 43°C. Protein aggregation was monitored by light scattering measurements, using a Varian spectrofluorometer. The excitation and emission wavelengths were set to 500 nm. The excitation and emission slit widths were set to 2.5 nm. Three independent experiments were performed.

#### Determination of the redox potential of HP0231 mutant proteins

The redox potentials of HP0231 mutant proteins were determined fluorometrically from the equilibrium constant with glutathione, as previously described (Roszczenko et al., 2012; Bocian-Ostrzycka et al., 2015b). The results are presented as the average of three independent experiments.

#### Oxidative folding of reduced RNaseA and refolding of scrambled RNaseA (scRNase)

*In vitro* oxidative folding of reduced, unfolded RNaseA (ruRNaseA) and refolding of scrambled RNaseA were performed with HP0231, HP0231 mutants and EcDsbA as described earlier (Bocian-Ostrzycka et al., 2015b).

### Phenotype assays—spot titers for cadmium resistance and motility assays

Spot titers for cadmium resistance and motility assays were performed to quantify the relative oxidase activity of HP0231 mutants *in vivo* as previously described (Ren et al., 2009; Bocian-Ostrzycka et al., 2015b).

## Results

### *In vivo* properties of the HP0231 with engineered catalytic motifs

*H. pylori* HP0231 was the first-described periplasmic dimeric oxidoreductase that has the physiological function of catalyzing disulfide formation (Roszczenko et al., 2012). Despite its structural resemblance to EcDsbG, the XX dipeptide from the active CXXC site of HP0231 is identical to that of EcDsbA (i.e., CPHC) but different from that of EcDsbC/G (i.e., CGYC/CPYC). Also, the *cis*-Pro loop of HP0231 is VcP, as in EcDsbA, where it is involved in DsbA-substrate(s) interaction. In contrast, there is a threonine residue found in the *cis*-Pro loop of EcDsbC and EcDsbG. It should also be noted that the catalytic domain of HP0231 is unusual. Structurally, it belongs to a class II DsbA, although it contains an active site characteristic for a class I DsbA (McMahon et al., 2014; Bocian-Ostrzycka et al., 2015b). Both the amino acid sequence of the CXXC motif and the *cis*-Pro loop influence the biochemical features of the Dsb proteins and, in consequence, determine their mode of action. Thus, we first investigated the effect of HP0231 catalytic motifs on their redox activity by generating several HP0231-mutated versions and analyzing their activity in living cells. Five versions of HP0231 were generated that mimic the EcDsbG and EcDsbC active sites. Two of them have catalytic motifs (CXXC and *cis*-Pro) identical to EcDsbG or EcDsbC (Table 3, positions 4 and 5, respectively). The CXXC motifs of two next variants correspond to those present in active sites of *E. coli* DsbG or DsbC, but are paired with native VcP instead of TcP motif (Table 3, positions 1 and 2, respectively). Additionally, a fifth HP0231 variant that has native CPHC motif combined with TcP was also generated (Table 3, position 3). The rationale for creating this variant was the observation that all the members of class II DsbAs that have been characterized so far, contain a TcP motif (McMahon et al., 2014). To investigate the significance of the cysteine residues of the CXXC motif, four HP0231 versions having cysteine residues replaced by alanine or serine were generated (Table 3, positions 6–9).

**Table 3:** **Mutated forms of HP0231used in this study**.

**No**.	**Mutation**	**CXXC/cisPro**	**Catalytic motif**	***In vitro*** **properties**					***In vivo E. coli*** **complementation**					***In vivo H. pylori hp0231***−**complementation**	
				**DsbG chaperone activity**		**DsbA oxidative activity**	**DsbC izomerizing activity**	**Reductase activity**	***dsbA−***			***dsbAB−***	***dsbC−***		
				**CS aggregation**	**Redox potential**	**ruRNase activity**	**scRNase activity**	**Insulin reduction**	**AP**	**Motility**	**CdCl_2_ resistance**	**Motility**	**Mucoid phenotype**	**Motility**	**CdCl_2_ resistance**
	HP0231	CPHC/VcP	DsbA/DsbA	+ +	−112 mV	+ +	−	+ +	+ + +	+ +	+	+ +	−	+ +	+
1	H161Y	CPYC/native	DsbG/native	+ + +	−112 mV	+	+	+ +	+ +	+ +	+	+	+	+	+
2	P160G, H161Y	CGYC/native	DsbC/native	+ + +	−121 mV	+	−	+ +	+ + +	+	+	−	+	+	+
3	V257T	native/TcP	native/DsbGC	+ +	−85 mV	+ +	−	+	−	−	−	−	−	−	−
4	H161Y, V257T	CPYC/TcP	DsbG	+ +	−90 mV	+ +	−	+	+ +	+	+	−	−	+	+
5	P160G, H161Y, V257T	CGYC/TcP	DsbC	+ +	−112 mV	+ +	−	+ +	+ +	+	+	−	−	+	+
6	C162S	CPHS	na	+ + +	nt	nt	nt	nt	−	−	nt	nt	−	−	−
7	C162A	CPHA	na	nt	nt	nt	nt	nt	+ + +	+	nt	nt	−	+	−
8	C159A	APHC	na	nt	nt	nt	nt	nt	−	−	nt	nt	nt	−	−
9	C159A, C162A	APHA	na	nt	nt	nt	nt	nt	−	−	nt	nt	nt	−	−

*CS, citrate synthase; ruRNase, reduced unfolded RNaseA; scRNaseA, scrambled RNase A; AP, alkaline phosphatase; nt, not tested; na, applicable*.

The *hp0231* gene, together with its own promoter, was cloned into pGEM-T Easy, and the designed mutations were introduced by site directed mutagenesis. Next, the DNA fragments encoding mutated versions of the *hp0231* were recloned into shuttle vectors pHel3 and pHel2 and successfully introduced into *H. pylori* and *E. coli* cells, respectively. The presence of HP0231-mutated forms in *H. pylori* and *E. coli* cells was confirmed by Western-blot experiments using rabbit specific anti-HP0231 serum (data not shown). All of the mutated forms, similar to native HP0231, function as homodimers, as shown by size exclusion (Supplementary Table S3).

**Figure 1:**
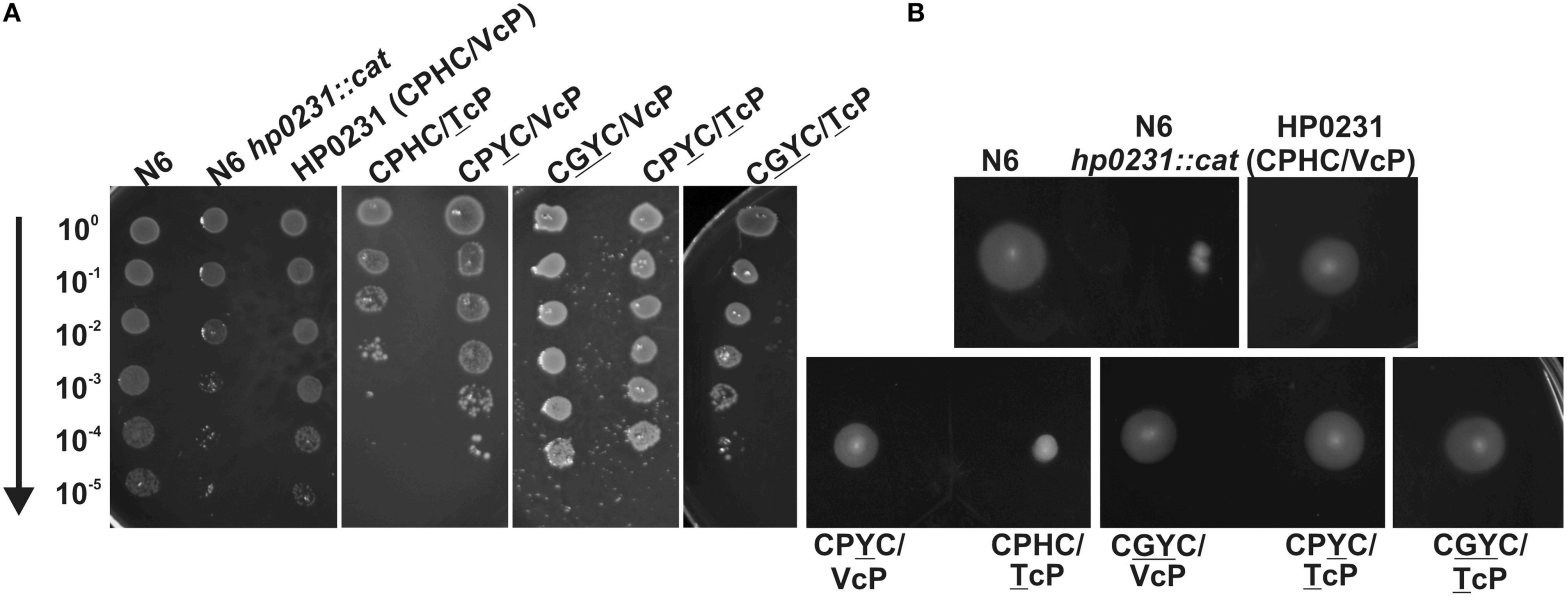
**HP0231 with CPHC/TcP is not active in *H. pylori hp0231::cat* cells**. As a positive control, *H. pylori N6 hp0231::cat* was transformed with pHel2 carrying the native *hp0231* gene with CPHC/VcP motifs. **(A)** Cadmium sensitivity assay. Exponentially growing *H. pylori* (wt, the *hp0231::cat* mutant and *hp0231::cat* complemented in trans with *hp0231* or its mutated forms). Cultures were 10-fold serially diluted, spotted on BA plates with 8 μM CdCl_2_, and incubated at 37°C. The mutant shows reduced growth after 3 days of incubation on plates containing cadmium chloride. All but one (HP0231 with CPHC/TcP) of the mutated forms, partially restored the cadmium resistance of the *H. pylori hp0231::cat*. **(B)** Motility assay. Bacterial motility was monitored after 4 days of incubation on 0.35% MH-agar plates containing 10% FCS. The *hp0231::cat* mutated strain and the same strain complemented in *trans* with TcP mutated form are non-motile.

To start, we examined the ability of the HP0231-mutated forms to complement an *hp0231* mutation by motility and cadmium resistance assays. Previously, we showed that *H. pylori* lacking HP0231 is non-motile; the mutated cells are flagellated as normal and the mechanism for non-motility remains unclear (Roszczenko et al., 2012). Cadmium is a divalent metal ion that is toxic, mainly due to its high affinity for thiol groups of proteins (Chrestensen et al., 2000; Quan et al., 2007). We found that all but one (CPHC/TcP) of the mutated HP0231 versions were active in disulfide bond generation in *H. pylori* to varying degrees, as shown by both assays. The HP0231 containing the CPYC/VcP motif presents a slightly lower oxidizing activity than the three other HP0231-mutated forms (Figures 1A,B).

**Figure 2:**
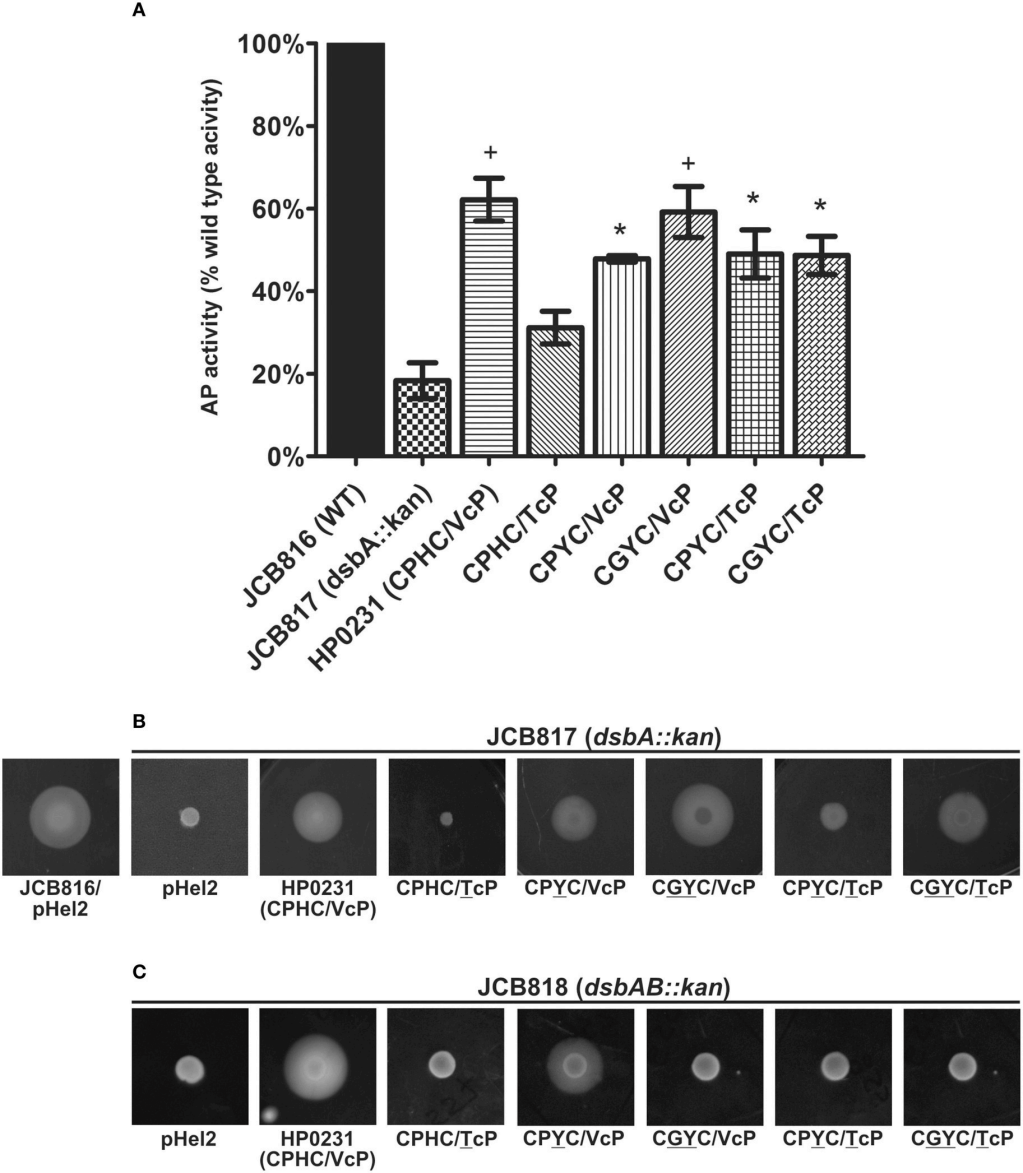
**Only HP0231 with the CPHC/TcP motifs did not restore the *E. coli dsbA::kan* wild type phenotype in two functional assays**. As negative controls, *E. coli dsbA::kan* was transformed with an empty pHel2 vector. **(A)** Alkaline phosphatase (AP) assay. The bars represent average activity of three independent experiments (*n* = 3) with the wild type set to 100% activity. There are significant differences (*p* < 0.001) in relative alkaline phosphatase activity between the *E. coli wt* cells and the *E. coli dsbA::kan* mutant strain, and also the strains complemented *in trans* by *hp0231* and *hp0231*-mutated forms. Error bars marked with asterisk (^*^) indicate no significant difference between *dsbA::kan* complemented with CPYC/VcP, CPYC/TcP and CGYC/TcP—these strains are slightly less active than strains with bars marked with plus sign (^+^); these indicate no significant difference between *dsbA::kan* complemented with native form of HP0231 and CGYC/VcP mutant (ANOVA followed by *post-hoc* Tukey's test). Alkaline phosphatase activity of wild type and *dsb* mutants and complemented strains was performed in M63 minimal medium. **(B)** Motility of the *E. coli dsbA::kan* complemented *in trans* by *hp0231*-mutated forms. Bacterial motility was monitored after 24 h of incubation on 0.35% LB-agar plates. The *E. coli dsbA::kan/*HP0231(CPHC/TcP) is non-motile, while *E. coli dsbA::kan/*CPYC/TcP is less motile than other strains. The figure presents a representative result. **(C)** Motility of the *E. coli dsbAB::kan* complemented *in trans* by *hp0231*-mutated forms. Only the *E. coli dsbA::kan/*CPYC/VcP can restore motility. The figure presents a representative result.

To shed more light on the impact of the HP0231 catalytic motif on protein oxidative folding, we also evaluated the ability of HP0231 variants to complement a DsbA or DsbC deficiency in *E. coli*, where a quite different Dsb network is operating. The *E. coli* strains lacking a functional DsbA exhibit a pleiotropic phenotype, including loss of motility and low alkaline phosphatase (AP) activity (Hatahet et al., 2014). Thus, we evaluated EcDsbA complementation by analyzing alkaline phosphatase (AP) activity (Figure 2A) and the recovery of cell motility (Figure 2B). The *E. coli* JCB817 (*dsbA*^−^) strain harboring the empty vector pHel2 is completely non-motile, and it displays only 20% of the AP activity of a wt strain. Similar to the results in *H. pylori* cells, all but one (CPHC/TcP) of the mutated versions of HP0231 were able to complement the Ec*dsbA* mutation. However, it should be noted that activity of this mutant was slightly higher than Ec*dsbA* mutant (20% vs. 30%) but still significantly lower than that of native HP0231 (30% vs. 60%). All four positive variants restored about 50% of wt *E. coli* AP activity (nearly the same level as native HP0231) and cell motility, though to varying degrees (Figures 2A,B). HP0231 functions in *E. coli* in an EcDsbB-independent manner. Thus, we asked whether the four HP0231-mutated versions were able to function irrespective of the presence of EcDsbB. To check this, we analyzed the motility of *E. coli dsbA*^−^*dsbB*^−^ strains harboring HP0231-mutated variants cloned on pHel2. We found that only HP0231 CPYC/VcP, acted in an EcDsbB-independent manner—similar the native HP0231. The other HP0231-mutated variants did not restore motility in *E. coli dsbA*^−^*dsbB*^−^ (Figure 2C).

**Figure 3:**
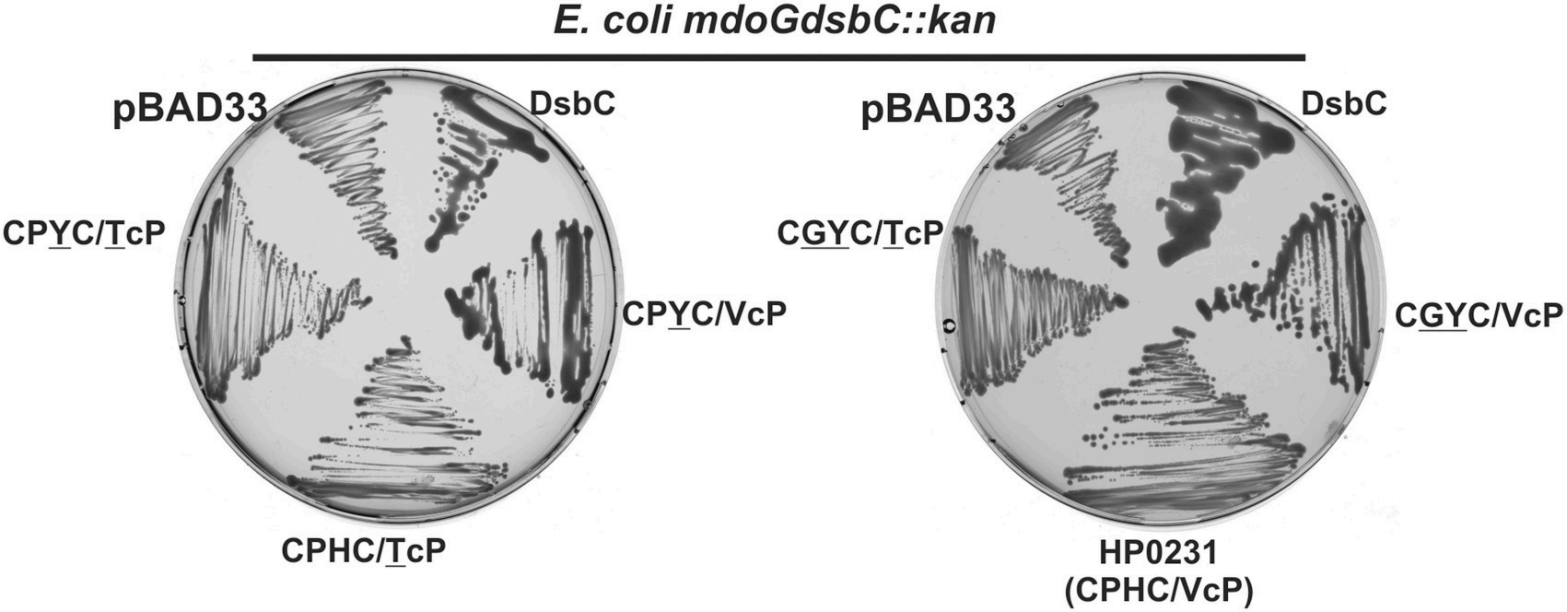
**Only two mutated forms of HP0231 (CPYC/VcP and CGYC/VcP) function as an isomerase in an *mdoGdsbC::kan* strain**. The *E. coli mdoGdsbC::kan* strain harboring various recombinant plasmids (pBAD33, pBAD33/DsbC^+^, pHel2/HP0231^+^, pHel2/HP0231-mutated forms) were grown on M63 minimal medium for 2 days at room temperature. The mucoid phenotype of the *mdoGdsbC*/DsbC^+^ strain was evaluated. OnlyHP0231 with CPYC/VcP or CGYC/VcP motifs complement the *dsbC* mutation.

Two of the HP0231 variants possess the CXXC motif of EcDsbC paired with TcP or VcP, and two have the CXXC motif of EcDsbG combined with TcP or VcP. DsbC from *E. coli* is required for *in vivo* copper (a redox-active metal) resistance, whereas EcDsbG is not involved in this activity (Hiniker et al., 2005, 2007). As we previously have shown, HP0231 is not able to complement the lack of EcDsbC, as measured by the copper sensitivity test (Roszczenko et al., 2012). Thus, it was interesting to examine whether the HP0231-mutated versions could act to isomerize proteins that are mis-oxidized by copper. To check this, we investigated their ability to complement an EcDsbC deficiency in the copper sensitivity assay. We found that none of the mutated forms of HP0231 were able to complement the deficiency of EcDsbC (data not shown). However, copper is known to catalyze the formation of non-native disulfide bonds in periplasmic proteins, and therefore the copper sensitivity assay measures the global effect of EcDsbC activity. In order to more directly evaluate the role of the HP0231 catalytic motif in EcDsbC complementation, we examined all five HP0231-mutated versions for specific isomerization activity by checking their influence on the oxidative folding of a specific protein, EcRcsF. EcRcsF is a small, outer-membrane lipoprotein which activates the Rcs phosphorelay upon envelope stress. RcsF contains two non-consecutive disulfide bonds, therefore depends on DsbC for proper folding (Cho et al., 2014). Use of an *E. coli mdoGdsbC* mutant convincingly assesses the specific isomerase activity of Dsb proteins *in vivo*, as mutation of the *mdoG* gene, involved in the synthesis of membrane-derived oligosaccharide, activates the Rcs system in an RcsF-dependent manner. An *mdoG* mutant displays a mucoid phenotype on M63 minimal medium, while a double *mdoGdsbC* mutant does not, due to lack of activation of the Rcs cascade in the absence of correctly folded DsbC (Leverrier et al., 2011). Interestingly, we found that two mutated versions of HP0231, containing the CXXC motifs of EcDsbG or EcDsbC paired with native EcDsbA/HP0231 VcP motif, were partially able to complement the lack of EcDsbC (Figure 3). These two mutated variants also retain their oxidizing activities, which means they are capable of both generating disulfide bonds and catalyzing disulfide bonds rearrangements.

HP0231 is present in its native host in an oxidized form (Roszczenko et al., 2012). In *E. coli dsbA*^−^,HP0231 is maintained as a mixture of oxidized and reduced forms, where the oxidized form is dominant (Bocian-Ostrzycka et al., 2015b). Given that two of the HP0231-mutated variants are bifunctional, at least in *E. coli*, we next checked the *in vivo* redox state of the analyzed proteins by AMS trapping technique. We found that all of them, like native HP0231, were maintained in an oxidized state *in vivo*, even though HP0231 with CPHC/TcP did not exhibit oxidizing activity *in vivo*. In *E. coli dsbA*^−^ and *E. coli dsbC*^−^ cells, the five HP0231 variants examined were sustained as a mixture of oxidized and reduced forms. However, the oxidized form is predominant. In *E. coli* the redox state of the HP0231-mutated forms were examined using MalPeg instead of AMS in order to increase the distance between reduced and oxidized protein forms. There were no significant differences between individual mutants in different hosts or strains (Supplementary Figure S4).

**Figure 4:**
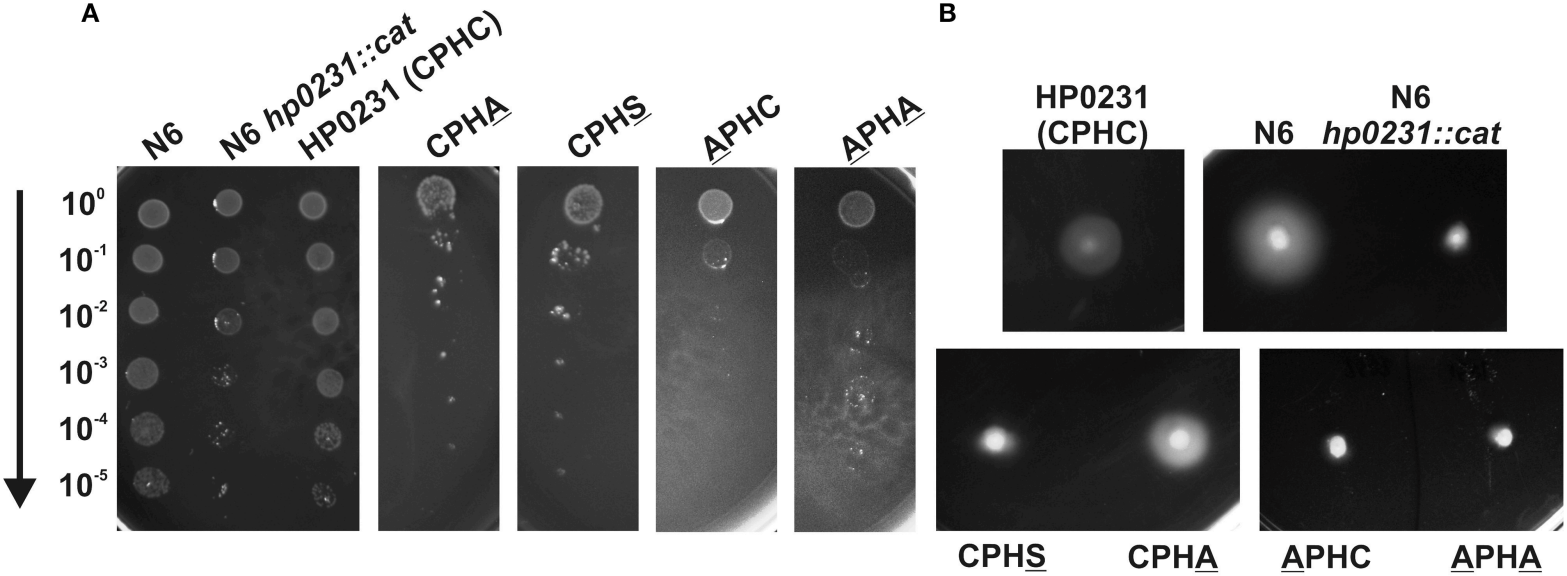
**HP0231 with CPHA/VcP motifs is active in *H. pylori hp0231::cat* cells**. As a positive control, *H. pylori N6 hp0231::cat* was transformed with pHel2 carrying the native *hp0231* gene. **(A)** Cadmium sensitivity assay. Exponentially growing *H. pylori* (wt, the *hp0231::cat* mutant and *hp0231::cat* complemented in trans with *hp0231* or its mutated forms). Cultures were 10-fold serially diluted, spotted on BA plates with 8 μM CdCl_2_, and incubated at 37°C. The mutant shows reduced growth after 3 days of incubation on plates containing cadmium chloride. Mutated forms having the cysteines of the CXXC motif changed to alanine or serine are inactive in this assay. **(B)** Motility assay. Bacterial motility was monitored after 4 days of incubation on 0.35% MH-agar plates containing 10% FCS. Only the *hp0231::cat* complemented in trans with HP0231 with CPHA/VcP is motile.

**Figure 5:**
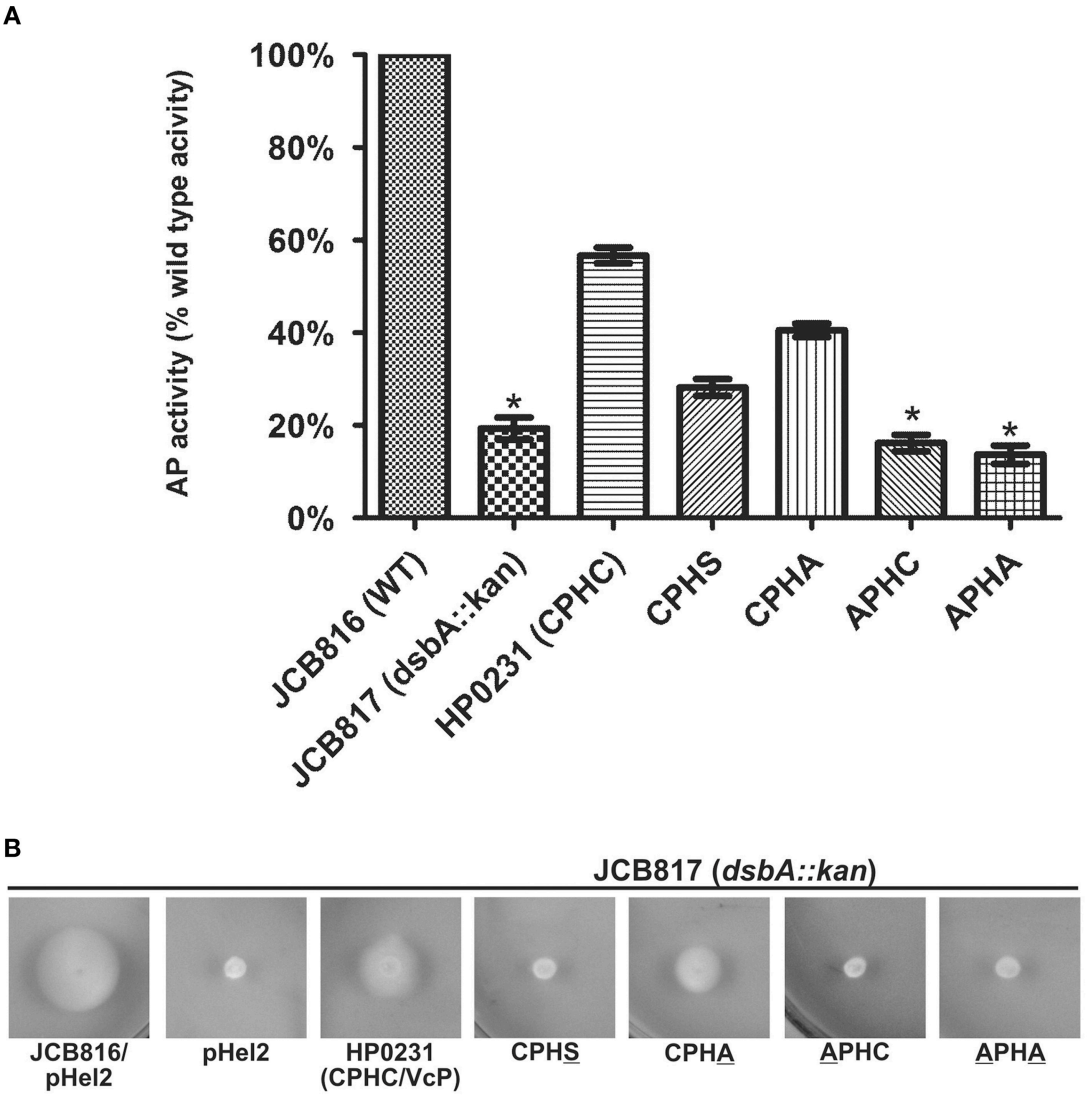
**HP0231 with the CPHA/VcP motifs is active in *E. coli dsbA::kan* cells. (A)** Alkaline phosphatase (AP) assay. The bars represent average activity of three independent experiments (*n* = 3) with the wild type set to 100% activity. There are significant differences (*p* < 0.001) in relative alkaline phosphatase activity between the *E. coli wt* cells and the *E. coli dsbA::kan* mutant strain, and also the strains complemented *in trans* by *hp0231* and *hp0231*-mutated forms. Error bars marked with an asterisk (^*^) indicate no significant difference between *dsbA::kan* complemented with APHC and APHA (ANOVA followed by *post-hoc* Tukey's test). Alkaline phosphatase activity of wild type and *dsb* mutants and complemented strains was performed in M63 minimal medium. **(B)** Motility of the *E. coli dsbA::kan* complemented *in trans* by *hp0231*-mutated forms. Bacterial motility was monitored after 24 h of incubation on 0.35% LB-agar plates. Only the *E. coli dsbA::kan* complemented with CPHA/VcP is motile. The figure presents a representative result.

To confirm the role of the HP0231 CXXC motif in the process of disulfide bond formation, four variants containing cysteine residues changed to alanine or serine were constructed (for details see Materials and Methods section). Three of the variants (APHC, CPHS and APHA) did not complement HP0231 deficiency, as measured by motility and cadmium resistance tests, illustrating the essential role of having two cysteine residues in the catalytic site for normal functioning (Figure 4). The activity of the HP0231 with CPHA was dependent on the test used. An *H. pylori hp0231* mutant with this version of HP0231 was cadmium sensitive, but its motility was restored. So, the activity of HP0231 with CPHA seems to be dependent on the target protein. It appears capable of generating disulfide bonds in specific proteins involved in motility, but it is not able to complement the global toxic effect of cadmium. A similar relation was seen when the HP0231 variants were examined in *E. coli* cells, using motility and AP activity tests. Three variants with the APHC, CPHS and APHA motifs do not complement an EcDsbA deficiency in either test. The HP0231 variant with the CPHA motif restores motility of *E. coli dsbA* mutant and restores AP activity at a level slightly lower than native HP0231 (Figures 5A,B).

Overall, the *in vivo* tests documented that all but one of the generated HP0231-mutated versions are involved in disulfide bond generation, both in *H. pylori* and *E. coli* cells. Additionally, two variants containing the EcDsbC or EcDsbG CXXC motif paired with VcP are bifunctional, with the ability to generate disulfide bonds as well as rearrange improperly introduced disulfide bonds.

### Biochemical characterization of mutated versions of HP0231

To shed more light on the role of the HP0231 catalytic motif and to confirm the data from the *in vivo* experiments, we studied the biochemical features of the HP0231-mutated versions. For biochemical experiments, the recombinant variants of HP0231 were purified from *E. coli* BL21 or Rosetta strains harboring appropriate recombinant plasmids.

**Figure 6:**
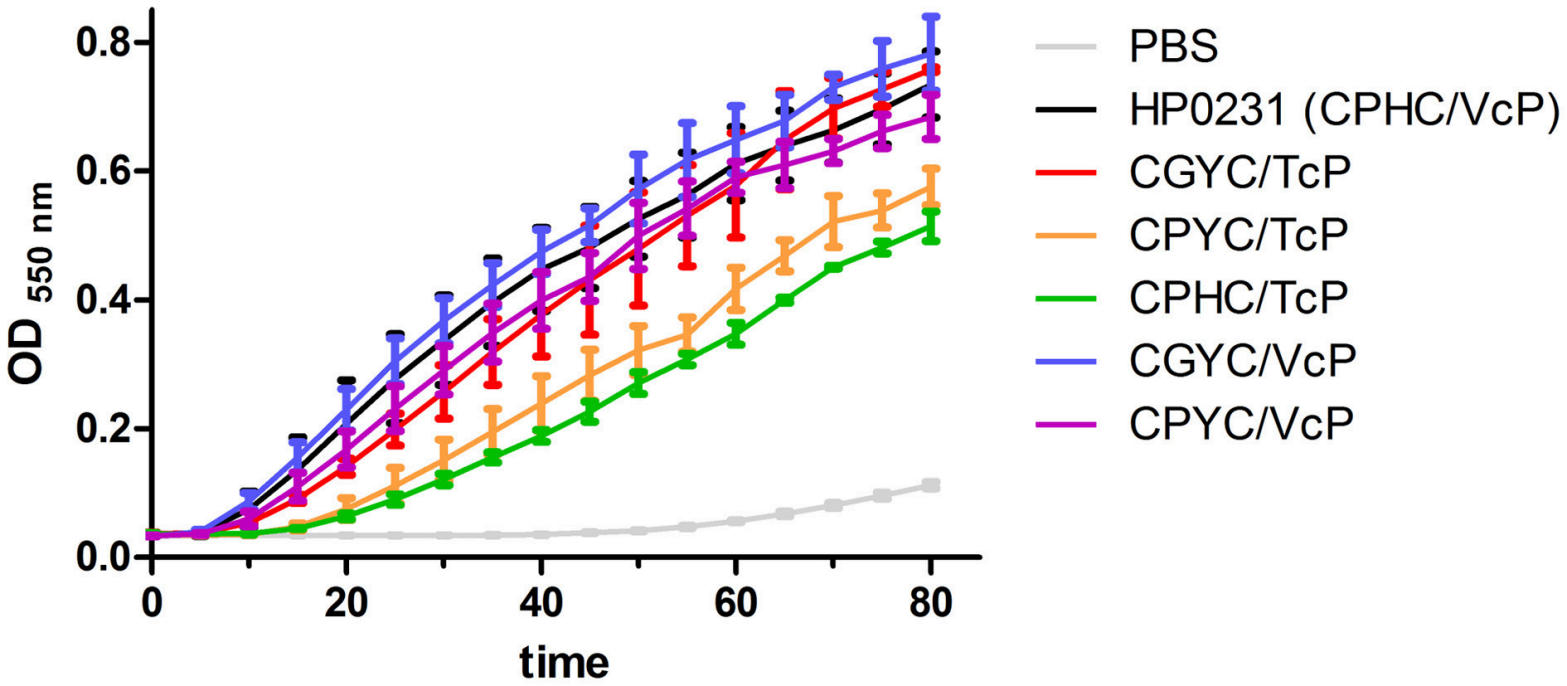
**Two HP0231-mutated versions (CPHC/TcP and CPYC/TcP) are less active in the insulin reduction assay**. The reaction contained 131 μM insulin in potassium phosphate buffer, pH 7.0 and 2 mM EDTA. The reaction was performed in the absence or presence of 10 μM EcDsbA and 10 μM HP0231-mutated forms. Reactions were started by adding DTT to a final concentration of 1 mM. Changes in the absorbance at 650 nm as a function of time were measured. The figure presents the average of three independent experiments (*n* = 3). Purified EcDsbA or HP0231 were used as a control.

We first determined the reductase activity of all mutated HP0231 forms by evaluating their ability to catalyze the reduction of insulin by DTT (Figure 6). This test is specific for disulfide oxidoreductases, and it defines reductase activity by the reduction of the intramolecular disulfide bond in the insulin. As previously shown, HP0231 acting as an oxidase displays activity that is slightly higher than EcDsbA but significantly lower than EcDsbC (Roszczenko et al., 2012). Two HP0231 mutated versions (CPHC/TcP and CPYC/TcP) are less active in the insulin reduction assay. The lowest level of activity in this test was observed for HP0231 with CPHC/TcP, the variant which that was inactive *in vivo*. Other HP0231 variants were able to reduce the insulin disulfide bond to a degree similar to that of native protein.

**Figure 7:**
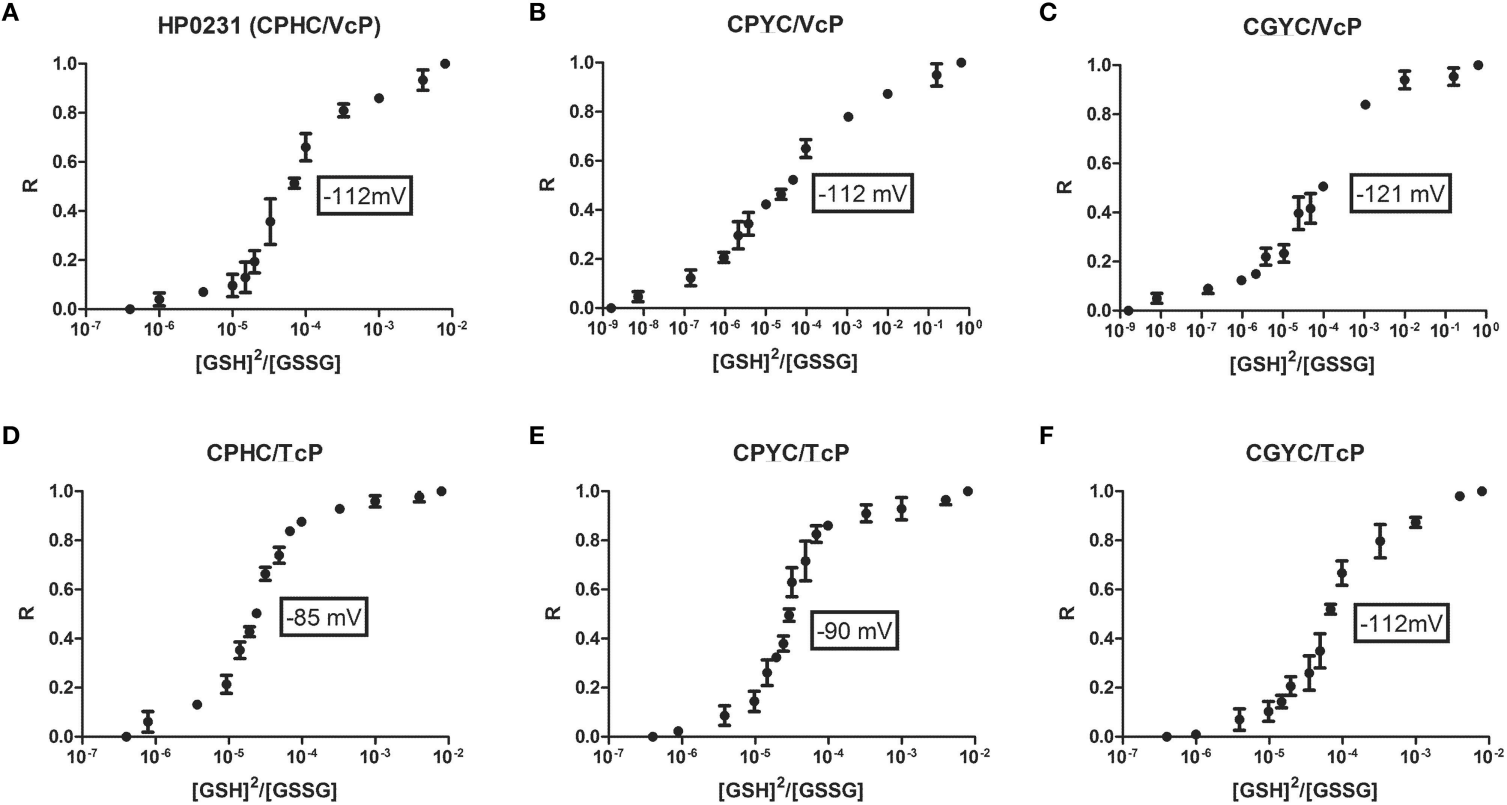
**(A96F)** The redox equilibrium of *H. pylori* HP0231-mutated forms with glutathione corresponds with their ability to reduce insulin in the insulin reduction assay. The fraction of reduced (R) HP0231-mutated forms was determined using the specific HP0231 fluorescence at 330 nm. The bars represent the average of three independent experiments.

Next, we determined the redox potentials of all mutated versions of HP0231 because the value of the redox potential reflects the activity of oxidoreductases (Figures 7A–F). The native HP0231 redox potential is similar to that of EcDsbA (−116 mV and −120 mV, respectively). Changing hydrophobic valine to more hydrophilic threonine in the *cis*-Pro loop resulted in an increase of its redox potential to −85 mV, making this protein more oxidizing. Similarly, the amino acid residue at *cis*-Pro minus 1 also influences the redox potentials of the other mutated HP0231 proteins. The redox potential of HP0231 (CPYC/TcP) was higher than that of HP0231 (CPYC/VcP) (−90 mV vs. −112 mV). Similar, though less noticeable, changes were observed for the variant having an EcDsbC CXXC motif (−112 mV for the variant with TcP vs. −121 mV for the variant with VcP). All these changes were consistent with activity of the recombinant proteins in the insulin reduction test (see above).

**Figure 8:**
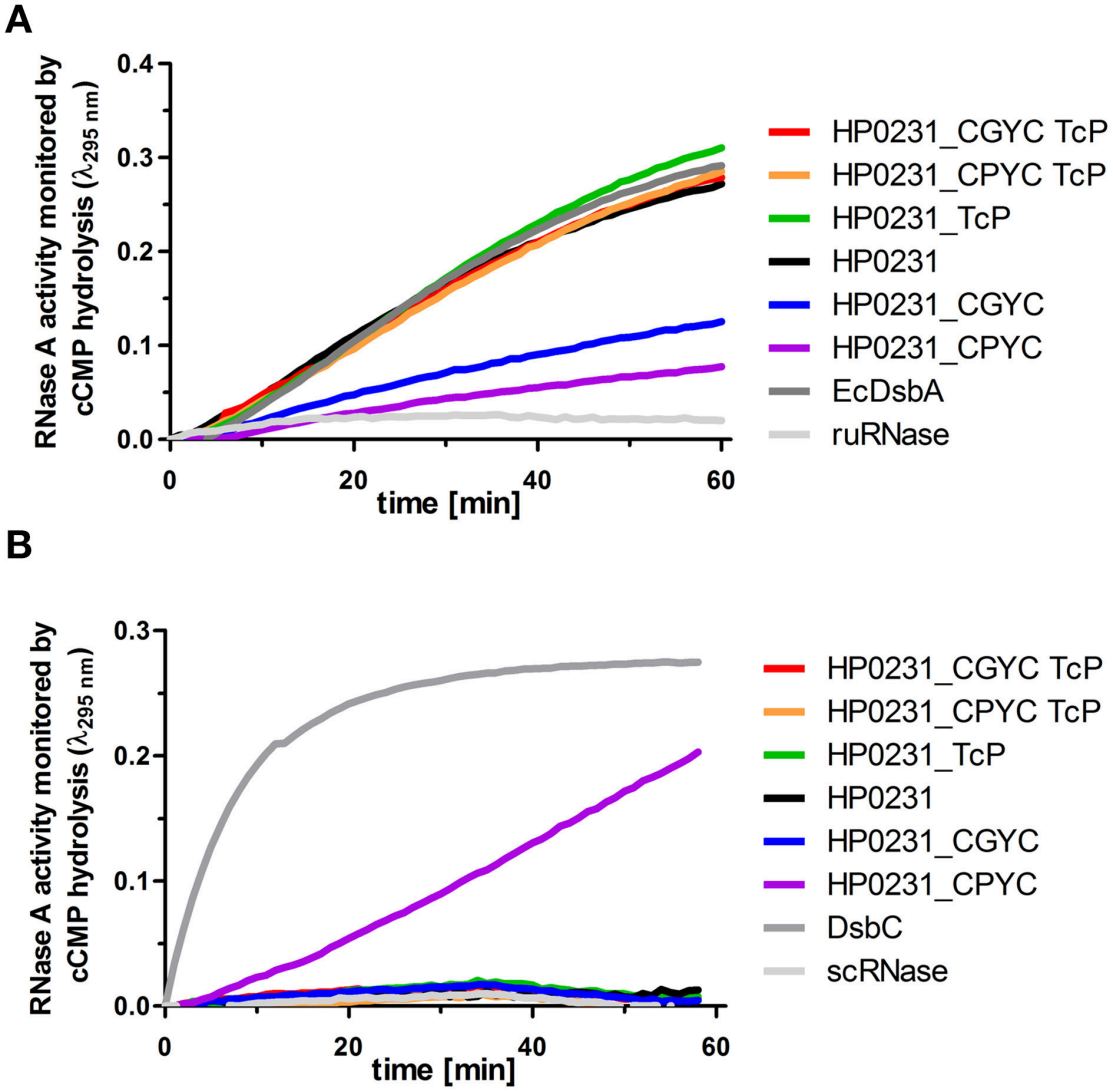
**RNase activity assays performed on purified HP0231 and HP0231-mutated forms**. Purified EcDsbA, EcDsbC or HP0231 were used as controls. **(A)** Two HP0231-mutated variants CPYC/VcP and CGYC/VcP are less active in an oxidase activity assay (reduced unfolded—ruRNase activity assay) compared to HP0231 wt and its other mutated forms. Reactions were carried out in 200 μl of PBS buffer containing 100 mM Tris acetate pH 8.0, 2 mM EDTA, 0.2 mM GSSG, 1 mM GSH, 4.5 mM cCMP, ruRNaseA (10 μM) and the analyzed enzyme (20 μM). The reaction was performed in the absence or presence of 20 μM EcDsbA, 20 μM HP0231, or 20 μM HP0231-mutated forms. Changes in absorbance at 296 nm as a function of time were measured. Three independent experiments were performed. The figure presents a representative result. **(B)** Only one HP0231-mutated variant (CPYC/VcP) functions as an isomerase in the scrambled RNase (scRNase) activity assay. Reactions were carried out in 200 μl of PBS buffer containing 100 mM Tris acetate pH 8.0, 2 mM EDTA, 10 μM DTT, 4.5 mM cCMP, scRNaseA (40 μM) and the analyzed enzyme (20 μM). Reactions were performed in the absence or presence of 20 μM EcDsbC, 20 μM HP0231 or 20 μM HP0231-mutated forms. Changes in absorbance at 296 nm as a function of time were measured. Three independent experiments were performed. The figure presents a representative result.

Next, to verify the observed *in vivo* oxidizing action of the analyzed recombinant proteins, we evaluated their ability to correctly oxidize reduced unfolded RNaseA (ruRNaseA), a protein with 4 disulfide bonds. Activity of HP0231 in this test is like that of EcDsbA (Bocian-Ostrzycka et al., 2015b). The results of this assay are given in Figure 8A. Unexpectedly, the HP0231-mutated version possessing a TcP motif, which was inactive in all *in vivo* tests, exhibits an oxidase activity that was even slightly higher than native HP0231. We also found that the two variants containing the CXXC motifs of EcDsbC or EcDsbG combined with TcP exhibited higher oxidase activities than their equivalents paired with VcP, implying a role for the threonine present in the *cis*-Pro loop in this oxidizing process.

Finally, we evaluated the ability of the HP0231-mutated versions to catalyze the isomerization of disulfide bonds using scrambled RNaseA (scRNaseA) as a substrate (Figure 8B). In its native form, RNase contains four disulfides that need to be properly rearranged. EcDsbC, a good catalyst of disulfide bond isomerization, was used as a positive control. HP0231 cannot catalyze this isomerization, and its activity resembles that of EcDsbA (Bocian-Ostrzycka et al., 2015b). We found that only the HP0231 with CPYC/VcP was able to reactivate scrambled RNaseA. None of the other mutated variants of HP0231 were active in this assay.

**Figure 9:**
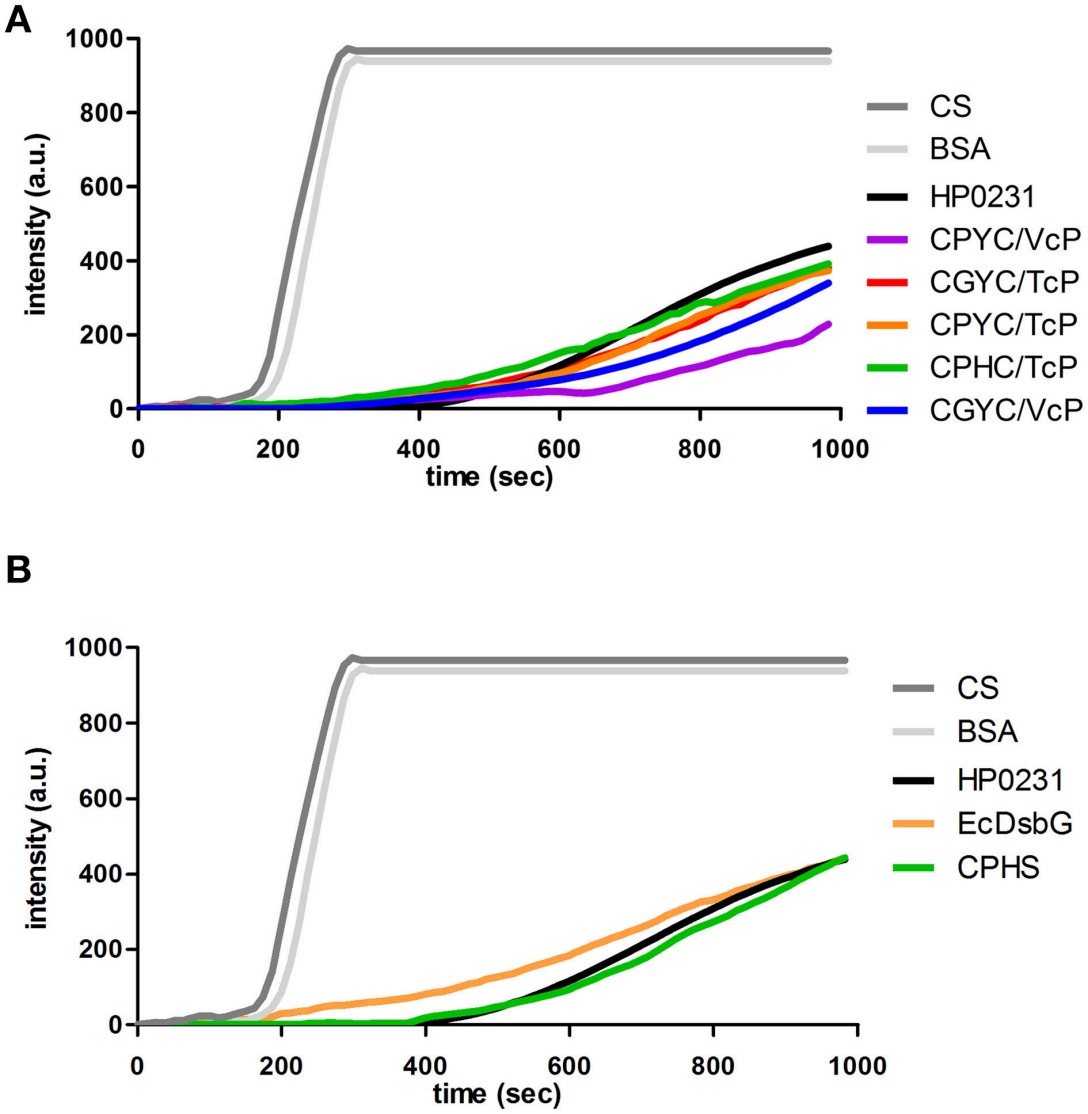
**(A,B)** All of the HP0231-mutated forms suppress the thermal aggregation of citrate synthase (CS) at 43°C at a similar level. 30 μM CS was diluted 200-fold into prewarmed 40 mM HEPES-KOH, pH 7.5, at 43°C in the absence or presence of 0.15 μM HP0231-mutated forms. Protein aggregation was monitored with light scattering measurements using a Varian spectrofluorometer. The excitation and emission wavelengths were set to 350 nm. The excitation and emission slit widths were set to 2.5 nm. To exclude non-specific protein effects, control experiments in the presence of 1.5 μM bovine serum albumin were conducted. **(A)** Chaperone activity of CXXC/XcP HP0231-mutated forms. **(B)** Chaperone activity of CPHS/VcP mutant. Three independent experiments were performed. The figure presents a representative result. Purified EcDsbG or HP0231 were used as a controls.

We and others have previously shown that the native HP0231, like the dimeric *E. coli* oxidoreductases EcDsbC and EcDsbG, functions as a molecular chaperone (Shao et al., 2000; Zhao et al., 2003). In the case of HP0231, however, its chaperone activity is not dependent on the presence of an N-terminal dimerization domain, as truncated HP0231 lacking this domain also acts as a strong chaperone (Bocian-Ostrzycka et al., 2015b). Thus, we also examined whether the HP0231-mutated versions function as molecular chaperones by checking their impact on the thermal aggregation of citrate synthase (Figure 9A). EcDsbG, a protein with high chaperone activity, was employed as positive control. HP0231, with its second cysteine replaced by serine in the CXXC motif (CPHS), was used to distinguish between chaperone and redox activities (Figure 9B). This variant was disfunctional in disulfide bond formation *in vivo* but remained active as chaperone, which indicates that chaperone activity is independent of the CXXC motif. Also, the HP0231 variant (CPHC/TcP), a protein defective in all the *in vivo* tests, functioned similarly to native HP0231. The two variants having the CXXC motifs of DsbC or DsbG paired with VcP were slightly more active in this assay than their equivalents with TcP.

Overall, the biochemical tests showed the critical role of the amino acid that precedes the proline in the *cis*-Pro loop. A hydrophilic threonine in this position makes the protein more oxidizing, compared to a hydrophobic valine, independently of the XX dipeptide present within CXXC motif. Although two of the HP0231-mutated versions (CPYC/VcP and CGYC/VcP) exhibited isomerase activity *in vivo* in *E. coli*, only the one with the CPYC/VcP motif was able to restore activity of scRNase *in vitro*.

### The influence of the dimerization domain on the activity in *H. pylori* and in *E. coli*

**Figure 10:**
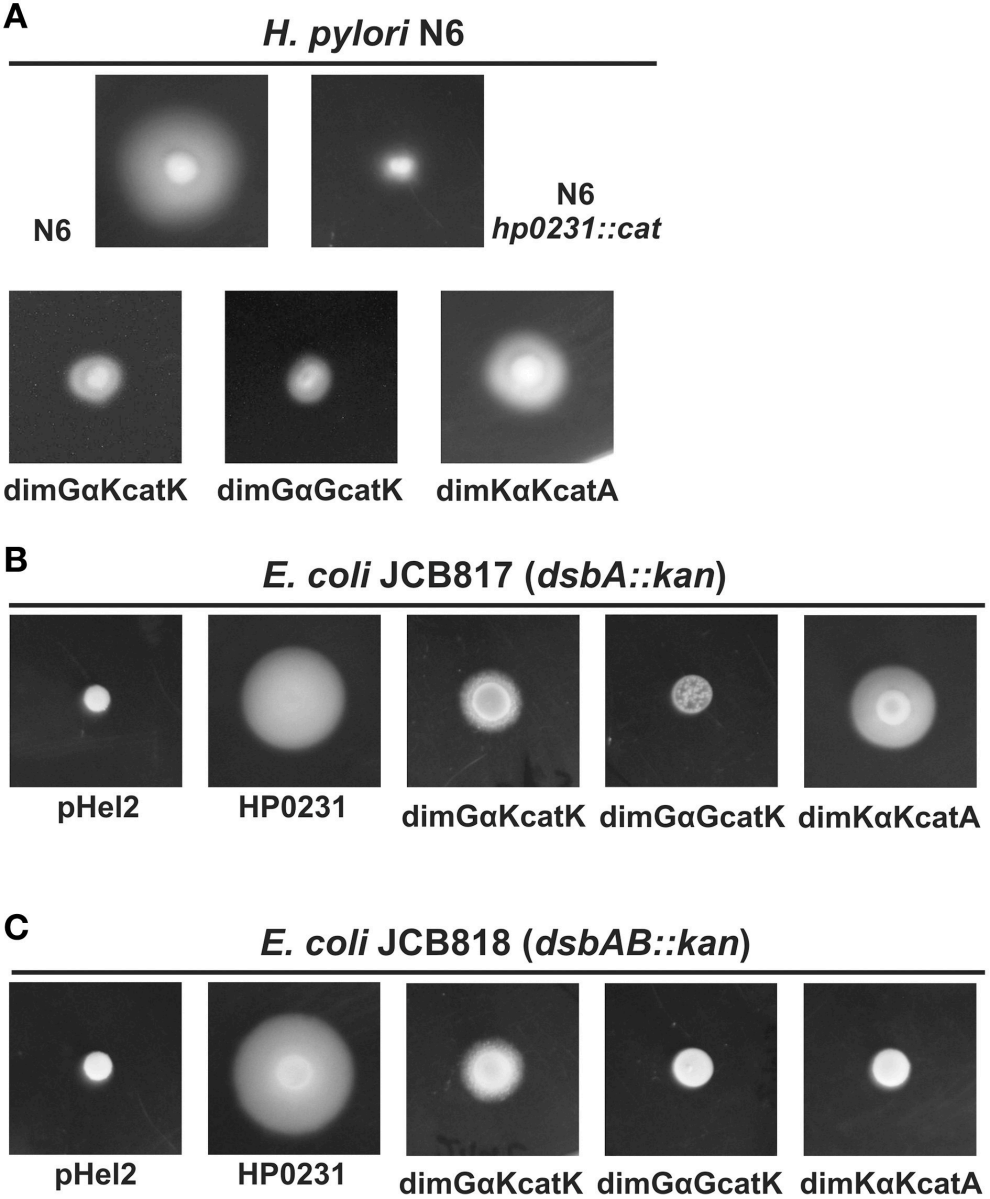
**In *H. pylori* N6 *hp0231::cat in vivo* complementation experiments only dimKαKcatA hybrid protein is active, but in EcDsbB-dependent manner. (A)** Motility assay of hybrid proteins. *H. pylori* motility was monitored after 4 days of incubation on 0.35% MH-agar plates containing 10% FCS. Only the *hp0231::cat*/dimKαKcatA strain is relatively motile. **(B,C)** *E. coli* motility was monitored after 24 h of incubation on 0.35% LB-agar plates. The *E. coli dsbA::kan/*dimKαKcatA is motile, in an EcDsbB-dependent manner, while two hybrids with DsbG dimerization domains are essentially non-motile.

HP0231 is an atypical dimeric oxidoreductase that plays a role in disulfide bond formation in *H. pylori*, as well as in *E. coli*. Its catalytic domain belongs to class II DsbA, which is rather characteristic for Gram-positive bacteria. The two classes of DsbA proteins are topologically distinct (McMahon et al., 2014; Bocian-Ostrzycka et al., 2015b). The HP0231 catalytic domain is specifically connected to the DsbG cluster; however it contains a catalytic motif characteristic for the class I DsbA. The HP0231 N-terminal dimerization domain is phylogenetically related to DsbC/G (Bocian-Ostrzycka et al., 2015b). Also, the helical linker of HP0231 joining the dimerization and catalytic domains is longer than that of EcDsbG (Yoon et al., 2011). Given all the atypical features of this protein, we asked the questions whether the dimerization domain of EcDsbG can act with the HP0231 catalytic domain, and whether the HP0231 dimerization domain can function with the catalytic domain of class I DsbA. Three fusion proteins were constructed (Table 2). The first is composed of the EcDsbG dimerization domain joined by the HP0231 linker to the HP0231 catalytic domain (chimera 1 in Table 2—dimGαKcatK). The second consists of the EcDsbG dimerization domain with its linker fused to the HP0231 catalytic domain (chimera 2 in Table 2—dimGαGcatK). The third contains the HP0231 dimerization domain with the HP0231 linker fused to the EcDsbA catalytic domain (chimera 3 in Table 2—dimKαKcatA). Precise descriptions of the chimeras, the details of their construction are described in the Materials and Methods section and in Table 2. Recombinant plasmids were introduced into *H. pylori hp0231*, as well as into *E. coli dsbA*^−^ and *E. coli dsbA*^−^*dsbB*^−^ strains. The correctness of all constructs was confirmed by sequencing. The presence of hybrid proteins in *H. pylori* and *E. coli* cells was confirmed by Western-blot experiments using rabbit specific anti-HP0231 serum or anti-His antibodies (Supplementary Figure S5). Oxidizing activity was monitored by the motility assay in both *H. pylori* and *E. coli*. We found that the two hybrid proteins (dimGαKcatK and dimGαGcatK)—composed of the EcDsbG dimerization domain with the EcDsbG or HP0231 linker, respectively, and the HP0231 catalytic domain—exhibited low activity in *H. pylori* cells (Figure 10A). DimKαKcatA chimera restored motility in both hosts. Interestingly, in contrast to native HP0231, its function in *E. coli* cells was EcDsbB-dependent (Figures 10B,C). Moreover, we found that none of the hybrid proteins were able to complement the deficiency of EcDsbC in a double *mdoGdsbC* mutant (data not shown). Together, these results demonstrate that the HP0231 dimerization domain is critical for protein activity in the native host, and that it cannot be substituted by the EcDsbG N-terminal domain. Interestingly, the data showed that the HP0231 dimerization domain does not constitute an obstacle for EcDsbB to reoxidize the EcDsbA catalytic domain present in hybrid protein, although EcDsbB does not react with native HP0231 (Roszczenko et al., 2012).

## Discussion

HP0231 of *Helicobacter pylori* was the first-described dimeric oxidoreductase involved in disulfide bond formation (Roszczenko et al., 2012). The list of identified dimeric oxidoreductases acting as oxidases is growing continuously. Apart from HP0231, the best characterized members of this group are *Legionella pneumophila* DsbA2 and *Francisella tulariensis* FtDsbA, also referred as FipB (Jameson-Lee et al., 2011; Qin et al., 2011, 2014). Even though LpDsbA2, FtDsbA, and HP0231 are all involved in disulfide bond formation, they differ considerably in many aspects. First of all, every analyzed bacterial species possesses distinct sets of Dsb proteins. The one and only similarity among them is the lack of the “classic” homolog of EcDsbC in their proteomes. The *L. pneumophila* genome encodes two DsbAs, two DsbBs and two DsbDs, whereas *F. tulariensis* possesses DsbA, DsbB and lacks DsbD (Kpadeh et al., 2013, 2015; Ren et al., 2014). In *E. coli* DsbD is responsible for DsbC re-reduction (Cho and Beckwith, 2009). Most *H. pylori* strains produce dimeric HP0231, which is the functional equivalent of DsbA, and DsbI (HP0595 in the *H. pylori* 26695 strain), the protein that is partially responsible for HP0231 reoxidation (Bocian-Ostrzycka et al., 2015a,b). Additionally, dimeric oxidoreductases of *L. pneumophila* and *F. tulariensis*, unlike HP0231, both have a bifunctional nature as they display both oxidase and isomerase activities (Qin et al., 2014; Kpadeh et al., 2015). To better understand the functioning of the *H. pylori* Dsb system, whose activity is required for full virulence, we focused on the functional and biochemical analysis of HP0231 point mutated versions, as well as on the similar analysis of hybrid fusion proteins of the EcDsbG and HP0231 dimerization domains with monomeric EcDsbA or the catalytic domain of HP0231.

First, using four mutated variants containing distinct CXXC motifs (APHA, APHC, CPHA, CPHS), we found that the CXXC motif present in the HP0231 thioredoxin domain is absolutely necessary for catalytic activity, as HP0231 variants having APHA, APHC, and CPHS are inactive in all *in vivo* tests, in both *H. pylori* and *E. coli* cells. We also noticed that HP0231 with a CPHA motif is active in some *in vivo* tests. In *H. pylori*, it complemented lack of native HP0231 in the motility test but not in the cadmium sensitivity assay. In *E. coli*, this HP0231 variant complemented lack of EcDsbA in both the motility test and the AP activity assay. Disulfide bond formation occurs in two stages. First, the N-terminal cysteine residues of the CXXC motifs, after a nucleophilic attack from a substrate protein, form an intermediate complex with a substrate protein. Second, the complex is resolved and the oxidized substrates released (Shouldice et al., 2011; Denoncin and Collet, 2013). Thus, it is expected that mutating the N-terminal cysteine residue of CPHC motif would completely abolish the protein activity. The observed difference of action between the two variants of HP0231 with CPHA or CPHS motifs is still unexplained. It should be noted that although, all so far characterized DsbA proteins contain CXXC motif, but some members of the thioredoxin fold class involved in the reduction of a substrate have CXXS or CXXT motif(Fomenko and Gladyshev, 2002, 2003; Atkinson and Babbitt, 2009).

The solved structure of HP0231 indicates that, out of two cysteine residues present in the CXXC motif of HP0231, only the first is solvent-exposed (Yoon et al., 2011). A possible explanation is that this single cysteine of the CPHA motif is oxidized to sulfenic acid, whereas the N-terminal cysteine residue present in the CPHS motif is protected against sulfenylation by serine, which is structurally similar to cysteine (Lo Conte and Carroll, 2013). However, activity of HP0231 variant with CPHA is substrate dependent. The similar observation has been made in term of mutated form of monomeric EcDsbA. Wunderlich et al. showed that EcDsbA containing CXXA motif acts as catalyst of oxidative protein in *in vitro* test (Wunderlich et al., 1995). The crystal structure of the CXXA EcDsbA mutant was solved and showed that this mutated form undergoes conformational changes and is able to form a dimer via one intermolecular disulfide bond between N-terminal cysteine residues. The authors speculated that the process is responsible for its interaction with EcDsbB. However, the activity *in vivo* of this form of EcDsbA has not been analyzed yet (Ondo-Mbele et al., 2005).

Our work shows the crucial role of the *cis*-Pro loop in modulating the oxidoreductase activities of HP0231. First of all, changing the hydrophobic valine to hydrophilic threonine in the HP0231 *cis*-Pro motif resulted in loss of its ability to create disulfide bonds, and the process was independent of the host background, as it was observed in both *H. pylori* and *E. coli* cells. However, biochemical tests revealed that HP0231 V257T possessed significantly more oxidizing redox potential compared to native variant (−85 mV vs. −116 mV) and less reducing activity in the insulin reduction assay. Furthermore, it is capable of oxidizing reduced, unfolded RNaseA as efficiently as native HP0231. Thus, our work shows that conclusions concerning Dsb protein function that draw from their biochemical attributes should be treated with caution. While their biochemical features are generally determined by their catalytic motifs and structure, their functioning *in vivo* is dependent on the composition of the Dsb network. *In vivo* functioning of oxidoreductases depends also on the redox conditions of the environment in which they act, as was shown by changing the location of cytoplasmic thioredoxin (Debarbieux and Beckwith, 1998, 2000). The similar lack of correlation between *in vivo* and *in vitro* features of EcDsbA V150T was noticed by Ren et al. and was interpreted as an inability to react with its upstream partner, EcDsbB (Ren et al., 2009). In the case of HP0231, these results should be interpreted with caution, as HP0231 oxidizing activity in *E. coli* is DsbB-independent, and in *H. pylori* it is moderately determined by the action of DsbI (HP0595), which is paralogous to the DsbB family (Raczko et al., 2005; Roszczenko et al., 2012). Although at this time we do not fully understand the mechanism, we hypothesize that changing V257T influences its ability to recognize downstream substrate/s and that the mode of action of this thiol oxidoreductase differs substantially in *E. coli* and *H. pylori*. Alternatively, changing the *cis*-Pro loop might influence the stability of the reduced form of this protein. The EcDsbA reduced form is more stable than the oxidized one. Thus, to be active, EcDsbA needs to be reoxidized by EcDsbB (Heras et al., 2008). In *E. coli*, HP0231 functions irrespective of the presence or absence of EcDsbB. Although we did not investigate this, we hypothesize that HP0231 is equally stable in its reduced and oxidized forms. Out of four analyzed HP0231-mutated forms that complement an EcDsbA deficiency, only the one with the CPYC/VcP motifs acts, like HP0231, in an EcDsbB-independent manner. The other three HP0231-mutated variants require EcDsbB reoxidation. So, it is highly probable that changing the catalytic motifs affects the stability of the HP0231 forms. This conjecture is corroborated by the fact that all of these exist in *E. coli* in mainly the oxidized form. Also, the similar stability of both forms of the DsbA from *Staphylococcus aureus* ensures its efficient functioning without DsbB (Heras et al., 2008). As HP0231 is the only described dimeric oxidoreductase involved in disulfide bond generation that is DsbB-independent, further biochemical and structural analysis of its mutated forms should be useful to understand its functioning.

As mentioned above, *H. pylori* does not encode an EcDsbC homolog. However, we previously demonstrated that HP0377 (CcmG playing a role in apocytochrome c reduction) confers *in vitro* a disulfide isomerase activity, and its functioning is related to HP0231 (Roszczenko et al., 2015). Thus, it may compensate the lack of DsbC homolog. As the two dimeric thiol-oxidoreductases, LpDsbA2 and FtDsbA, are bifunctional proteins with both oxidizing and isomerizing activity, we asked the question: which features of HP0231 prevent it from functioning in the isomerization pathway? We found that changing the catalytic motif to that of EcDsbC or EcDsbG (CPHC to CGYC or CPYC, respectively) and leaving the *cis*-Pro motif intact (VcP) generated bifunctional proteins. These two variants still retained their oxidizing activities, and at the same time, they acquired the ability to act as isomerases in *in vivo* tests. Also, in the case of the two described mutated forms of HP0231, the crucial role of the *cis*-Pro loop was clear, as their equivalents with a TcP motif were not active as isomerases in both *E. coli* and *H. pylori*. Furthermore, two of the HP0231-mutated forms (CPYC/VcP and CGYC/VcP) possess more reducing redox potential, exhibit lower oxidase activities and higher activities in the insulin reduction assay than their equivalents paired with TcP, which confirms their functions observed *in vivo*. However, only the HP0231 with CPYC/VcP was able to restore scRNase activity *in vitro*, indicating that the protein function is also determined by available substrate.

Chaperone activity has so far been attributed to homodimeric oxidoreductases such as EcDsbC or EcDsbG (Chen et al., 1999; Shao et al., 2000). Monomeric EcDsbA exhibits low chaperone activity *in vitro*, but chimeras of the N-terminal domain of EcDsbC with catalytic EcDsbA function *in vitro* as chaperones (Zhao et al., 2003; Segatori et al., 2004). The catalytic CXXC motif of EcDsbC is not necessary for its chaperone activity, as was shown by Liu and Wang (Liu and Wang, 2001). Thus, we tested whether the HP0231 CPHC/VcP catalytic motifs are necessary to prevent thermal aggregation of citrate synthase. Our data are consistent with those presented by Liu and Wang, as all of the HP0231-mutated versions, including the one with the CPHS motif, function as chaperones. Moreover, we previously have shown that a monomeric truncated version of HP0231 lacking the dimerization domain, in contrast to EcDsbA, displays high chaperone activity (Bocian-Ostrzycka et al., 2015b). Thus, we concluded that HP0231 chaperone activity requires neither enzyme dimerization nor the active catalytic motif. Our data are in agreement with those presented by Schmidt et al. concerning FtDsbA (Schmidt et al., 2013).

Previously, we showed that the HP0231 truncated version (class II DsbA) is active in disulfide bond formation in a DsbB-dependent manner, similar to EcDsbA in *E. coli* cells, but that it is inactive in *H. pylori*. Similarly, monomeric EcDsbA (class I DsbA) does not function in *H. pylori* (Bocian-Ostrzycka et al., 2015b). Thus, to shed more light on the function of the HP0231 dimerization domain, several chimeras were constructed. Our phylogenetic analysis showed that the HP0231 dimerization domain is related to the DsbG/C dimerization domains (Bocian-Ostrzycka et al., 2015b). So, we evaluated the functioning of the HP0231 catalytic domain fused to the EcDsbG dimerization domain with its own, or the HP0231, α-linker. Both variants displayed only moderate activity in *H. pylori* and did not function in *E. coli*, indicating that the EcDsbG dimerization domain cannot efficiently substitute for the HP0231 dimerization domain. We assume that the reason lies in the primary structure of the dimer cleft. The V-shaped cleft of *H. pylori* HP0231 exhibits different characteristics than the clefts of DsbC and DsbG, thereby suggesting different substrate specificity. The major difference is the surface charge. The inner surface of the *H. pylori* HP0231 model is lined with positive residues (i.e., Lys-41, Lys-45, Lys-46, Arg-48, Lys-72, Lys-97, Arg-238, Lys-246, Lys-247, Lys-255, and Lys-264). Moreover, there are also Asp-68, Asp-70, Asp-99, Asp-100, Glu-242, Glu-250 negative residues lining the presumed binding cleft). In contrast, *E. coli* DsbG has several acidic residues (i.e., Glu-11, Asp-36, Glu-69, Glu-79, Glu-189, Asp-193, and Asp-220), and *E. coli* DsbC has hydrophobic and uncharged residues (Heras et al., 2004). The positive residues form patches that are absent in both *E. coli* DsbC and DsbG. EcDsbA, which was inactive as monomer, complemented an *H. pylori hp0231*^−^ mutation, when fused to the N-terminal domain of HP0231, as measured by the motility test. These data indicate that the process of disulfide bond formation in *H. pylori* is conditioned by tertiary protein structure. Our data are generally consistent with studies on DsbC-DsbA chimera fusions, where these homodimers were found to interact with DsbB in the process of disulfide bond generation in *E. coli* (Segatori et al., 2004).

## Author contributions

EJ-K, KB-O, and AŁ conceived and designed the study. KB-O, MG, AB, KJ, KP, and AK carried out the laboratory work. EJ-K, KB-O, and AŁ analyzed the data. EJ-K, KB-O and JFC wrote the manuscript. All authors read and approved the final manuscript.

### Conflict of interest statement

The authors declare that the research was conducted in the absence of any commercial or financial relationships that could be construed as a potential conflict of interest.
