# Supplementary material for: Engineering of Helicobacter pylori Dimeric Oxidoreductase DsbK (HP0231)
Source: Front Microbiol. 2016 Jul 26;7:1158. doi: 10.3389/fmicb.2016.01158 (PMC4960241; doi:10.3389/fmicb.2016.01158)
Supplement: Supplementary file 1 [file DataSheet1.pdf]

Supplementary information

Engineering of *Helicobacter pylori* dimeric oxidoreductase DsbK (HP0231)

Katarzyna M. Bocian-Ostrzycka[1], Magdalena Grzeszczuk[1], Anna M. Banaś, Katarzyna Jastrząb, Karolina Pisarczyk, Anna M. Kolarzyk, Anna M. Łasica[1*], Jean-Francois Collet[2ab], Elżbieta K. Jagusztyn-Krynicka[1#]

Running title: Engineering of *H. pylori* HP0231 oxidoreductase.

Keywords: *Helicobacter pylori*, disulfide bonds, Dsb proteins, oxidoreductase, chaperone activity, site-directed mutagenesis, protein engineering

Affiliations:
[1] Department of Bacterial Genetics, Institute of Microbiology, Faculty of Biology, University of Warsaw, Warsaw, Poland
[2a] WELBIO, Avenue Hippocrate 75, 1200 Brussels, Belgium
[2b] de Duve Institute, Université catholique de Louvain (UCL), Avenue Hippocrate 75, 1200 Brussels, Belgium

[#] Corresponding author
E. Katarzyna Jagusztyn-Krynicka
Department of Bacterial Genetics, Institute of Microbiology, Faculty of Biology, University of Warsaw; Miecznikowa 1 str. 02-096 Warsaw, Poland
Phone: +4822 5541404; Fax: +4822 5541402

Email addresses:
EKJK: kjkryn@biol.uw.edu.pl; KMBO: kasia.bocian@gmail.com;
AML: alasica@biol.uw.edu.pl; MG: grzeszczuk.magdalena@gmail.com;
JFC: jfcollet@uclouvain.be

[*] Current address: Department of Oral Immunology and Infectious Diseases, University of Louisville, School of Dentistry, Louisville, KY 40202, USA

Table S1.Primers used in this study

	Name	Sequence 5'-3'	Orientation	Restriction site/mutation
	Primers used to create purification vectors			
1	231expI	GAGGCCATGGCTAATGACAAACGGATGCAG	forward	NcoI
2	231expII	GTGCTCGAGTGCCTTATAATGGTATAAGAA	reverse	XhoI
	Primers used to create hybrid genes			
3	HP231_BamL	GTGGGATCCGCCTGCTCTTCATCAATAACTTTAG	forward	BamHI
4	HP231prom_dsbg	CTGGAGCAGGAAGGTCATTAGCTGAAAC	reverse	Ø
5	dimDsbG_231prom	GTTTCAGCTAATGACCTTCCTGCTCCAG	forward	Ø
6	dimDsbG_231_alfa	CTAATTGCACATCATCGCTTTTTTCACCTTTCTCGTTGTAC	reverse	Ø
7	HP231_alfa_dsbg	GTACAACGAGAAAGGTGAAAAAAGCGATGATGTGCAATTAG	forward	Ø
8	dim_alfaDsbG_231kat	CGCATAATCAGCCGGTATGGATTGTTCCATCCGTTG	reverse	Ø
9	HP231kat_alfaDsbG	CAACGGATGGAACAATCCATACCGGCTGATTATGCG	forward	Ø
10	HP231 His_XhoR3	CAGAAATCTCGAGTCAATGGTGATGATGGTGATGATGGTGATGATGTGCCTTATAATGGTATAAGAAAGG	reverse	XhoI
11	HP0231dim_DsbA	CCATCTTCATACTGCGCTTCATTAAAAATAGCG	reverse	Ø
12	DsbAkat_231	CGCTATTTTTAATGAAGCGCAGTATGAAGATGG	forward	Ø
13	DsbAkat_HisXho	CGACTCGAGCTAGTGGTGGTGGTGGTGGTGTTTTTTCTCGGACAG	reverse	XhoI
	Primers used in site-directed mutagenesis			
14	Hp231C2SF	GATCCCATGTGCCCACATAGCCAAAAAGAGCTCAC	forward	C162S
15	Hp231C2SR	GTGAGCTCTTTTTGGCTATGTGGGCACATGGGATC	reverse	C162S
16	Hp231CA1	GATCCCATGTGCCCACATGCCCAAAAAGAGCTCAC	forward	C162A
17	Hp231CA2	GTGAGCTCTTTTTGGGCATGTGGGCACATGGGATC	reverse	C162A
18	HP231C2AmutL	TATTGTCTCTGATCCCATGGCCCCACATTGCCAAAAAGAG	forward	C159A
19	HP231C2AmutR	CTCTTTTTGGCAATGTGGGGCCATGGGATCAGAGACAATA	reverse	C159A
20	AXXA231F	CTTTATATTGTCTCTGATCCCATGGCCCCACATGCCCAAAAAGAGCTCACTAAACTTAG	forward	C159A, C162A
21	AXXA231R	CTAAGTTTAGTGAGCTCTTTTTGGGCATGTGGGGCCATGGGATCAGAGACAATATAAAG	reverse	C159A, C162A

	Name	Sequence 5'-3'	Orientation	Restriction site/mutation
22	HP231H-YmutL	CTGATCCCATGTGCCCATATTGCCAAAA AGAGCTC	forward	H161Y
23	HP231H-YmutR	GAGCTCTTTTTGGCAATATGGGCACATG GGATCAG	reverse	H161Y
24	HP231PH-GYmutL	ATTGTCTCTGATCCCATGTGCGGATATTG CCAAAAAGAGCTCACTA	forward	P160G, H161Y
25	HP231PH-GYmutR	TAGTGAGCTCTTTTTGGCAATATCCGCAC ATGGGATCAGAGACAAT	reverse	P160G, H161Y
26	HP231V-Tmut_R	AAGATTTTTGAATCTGGCGTGATTAAGG GTACGCCTTTCTTATACCATTAT	forward	V257T
27	HP231V-Tmut_L	ATAATGGTATAAGAAAGGCGTACCCTTA ATCACGCCAGATTCAAAAATCTT	reverse	V257T

Table S2. Plasmids used in this study

	Plasmids:		
1	pET28a	Km^r, IPTG inducible	Novagen
2	pGEM T-Easy	Ap^r; LacZα	Promega
3	pJET1.2	CloneJET PCR Cloning Kit; Ap^r	ThermoFisher Scientific
4	pHel2	Cm^r *E. coli/H. pylori* shuttle vector	(Heuermann and Haas, 1998)
5	pHel3	Km^r *E. coli/H. pylori* shuttle vector	(Heuermann and Haas, 1998)
6	pUWM389	$hp0231^+$ in pGEM T-Easy	(Roszczenko et al., 2012)
7	pUWM397	$hp0231^+$ in pHel3	(Roszczenko et al., 2012)
8	pUWM500	$hp0231^+$ in pHel2	(Roszczenko et al., 2012)
9	pUWM513	$hp0231_{CXXS}{}^+$ (C162S) in pHel3	This study
10	pUWM531	$hp0231_{CXXS}{}^+$ (C162S) in pHel2	This study
11	pUWM517	$hp0231_{CXXA}{}^+$ (C162A) in pHel3	This study
12	pUWM530	$hp0231_{CXXA}{}^+$ (C162A) in pHel2	This study
13	pUWM2031	$hp0231_{AXXC}{}^+$ (C159A) in pHel3	This study
14	pUWM2058	$hp0231_{AXXC}{}^+$ (C159A) in pHel2	This study
15	pUWM2032	$hp0231_{AXXA}{}^+$ (C159A, C162A) in pHel3	This study
16	pUWM2060	$hp0231_{AXXA}{}^+$ (C159A, C162A) in pHel2	This study
17	pUWM545	$hp0231_{CPYC}{}^+$ (H161Y) in pHel3	This study
18	pUWM560	$hp0231_{CPYC}{}^+$ (H161Y) in pHel2	This study
19	pUWM572	$hp0231_{CGYC}{}^+$ (P160G, H161Y) in pHel3	This study
20	pUWM558	$hp0231_{CGYC}{}^+$ (P160G, H161Y) in pHel2	This study
21	pUWM573	$hp0231_{CPYC/TcP}{}^+$ (H161Y, V257T) in pHel3	This study
22	pUWM559	$hp0231_{CPYC/TcP}{}^+$ (H161Y, V257T) in pHel2	This study
23	pUWM580	$hp0231_{CGYC/TcP}{}^+$ (P160G, H161Y, V257T) in pHel3	This study
24	pUWM579	$hp0231_{CGYC/TcP}{}^+$ (P160G, H161Y, V257T) in pHel2	This study
25	pUWM546	$hp0231_{TcP}{}^+$ (V257T) in pHel3	This study
26	pUWM557	$hp0231_{TcP}{}^+$ (V257T) in pHel2	This study

27	pUWM2115	translational fusion of fragments encoding: dimerization domain of EcDsbG, α-linker and catalytic domain of HP0231 under native *hp0231* promoter in pHel3	This study
28	pUWM2130	translational fusion of fragments encoding: dimerization domain of EcDsbG, α-linker and catalytic domain of HP0231 with native *hp0231* promoter in pHel2	This study
29	pUWM2116	translational fusion of fragments encoding: dimerization domain and α-linker of EcDsbG, and catalytic domain of HP0231 with native *hp0231* promoter in pHel3	This study
30	pUWM2131	translational fusion of fragments encoding: dimerization domain and α-linker of EcDsbG, and catalytic domain of HP0231 with native *hp0231* promoter in pHel2	This study
31	pUWM2117	translational fusion of fragments encoding: dimerization domain and α-linker of HP0231, and catalytic domain of EcDsbA with native *hp0231* promoter in pHel3	This study
32	pUWM2132	translational fusion of fragments encoding: dimerization domain and α-linker of HP0231, and catalytic domain of EcDsbA with native *hp0231* promoter in pHel2	This study
33	pUWM525	$hp0231^{+}$ in pET28a	(Roszczenko et al., 2012)
34	pUWM2062	$hp0231_{CXXS}{}^{+}$ (C162S) in pET28a	This study
35	pUWM2061	$hp0231_{CXXA}{}^{+}$ (C162A) in pET28a	This study
36	pUWM2103	$hp0231_{CPYC}{}^{+}$ (H161Y) in pET28a	This study
37	pUWM2084	$hp0231_{CGYC}{}^{+}$ (P160G, H161Y) in pET28a	This study
38	pUWM2038	$hp0231_{CPYC/TcP}{}^{+}$ (H161Y, V257T) in pET28a	This study
39	pUWM2039	$hp0231_{CGYC/TcP}{}^{+}$ (P160G, H161Y, V257T) in pET28a	This study
40	pUWM2040	$hp0231_{TcP}{}^{+}$ (V257T) in pET28a	This study
41	pET28a/*EcdsbA*	$EcdsbA^{+}$ in pET28a	JFC Collection
42	pET28a/*EcdsbC*	$EcdsbC^{+}$ in pET28a	JFC Collection
43	pET28a/*EcdsbG*	$EcdsbG^{+}$ in pET28a	JFC Collection

Table S3. Size exclusion profiles of the purified *H. pylori* proteins separated on an ENRich SEC70 column (Bio-Rad) and monitored by absorbance at 280 nm. Native HP0231 elutes as a single peak at 9.15 min, with an estimated mass of 56 kDa, consistent with the size of the homodimer.

Protein	Time of elution
**native HP0231**	9.15 min
**HP0231 CPYC/VcP**	9.22 min
**HP0231 CGYC/VcP**	9.16 min
**HP0231 CPHC/TcP**	9.21 min
**HP0231 CPYC/TcP**	9.18 min
**HP0231 CGYC/TcP**	9.16 min
**HP0231 CPHS**	9.15 min
**HP0231 CPHA**	9.20 min

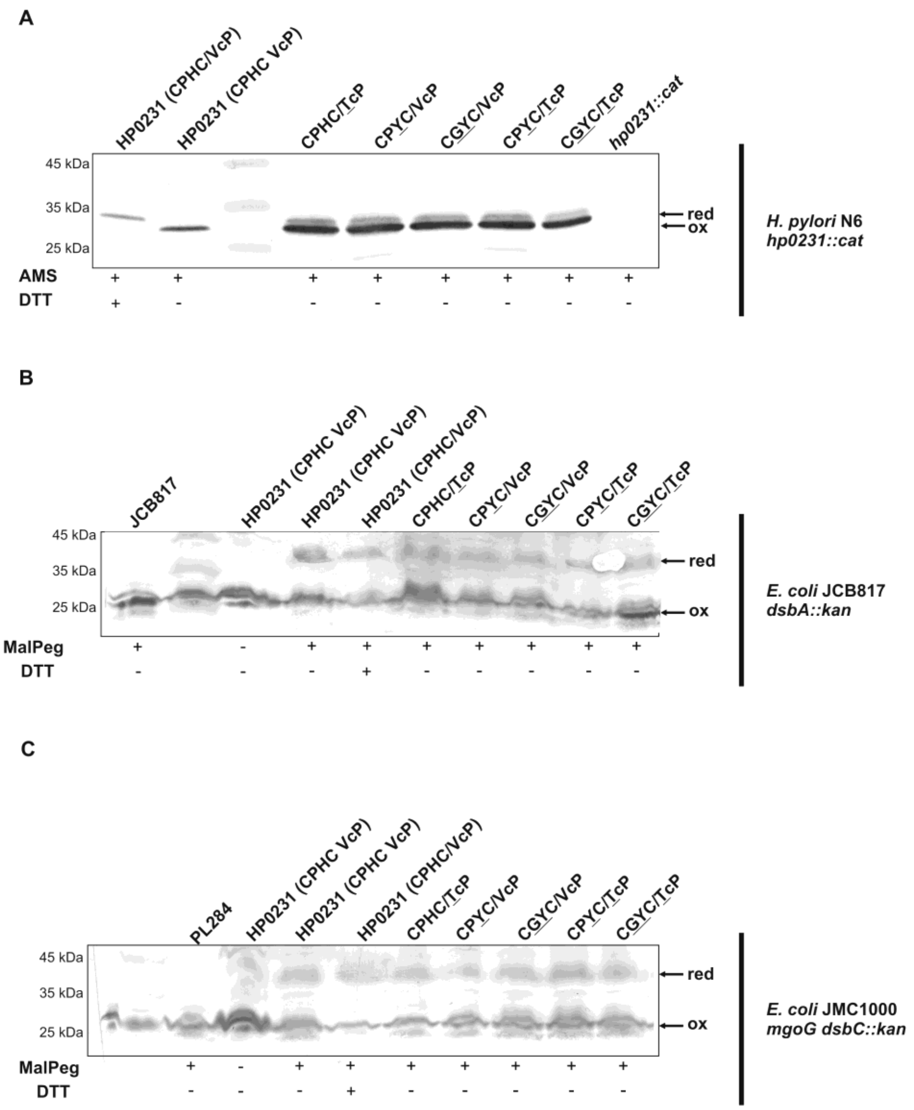

Figure S4.
Redox state of HP0231 mutated forms in *E. coli* and in *H. pylori*. **Panel A**: HP0231 mutated forms in *H. pylori N6 hp0231::cat* mutants modified with AMS. **Panel B**: HP0231 mutated variants in *E. coli dsbA::kan*. **Panel C**: *E. coli dsbA::kan* modified with MalPEG. Bacterial cultures were treated with 10% (v/v) TCA, followed by alkylation with AMS or MalPEG. Cellular proteins including the reduced (red; DTT treated, modified with AMS or MalPEG) and the oxidized (ox; non-modified with AMS/MalPEG) controls were separated by 18% (for AMS) or 12% (for MalPEG) SDS-PAGE under non-reducing conditions, followed by Western-blot analysis using rabbit antibodies against HP0231. Each lane contains proteins solated from the same amount of bacteria. In panels B and C the reduced form was visible only when the gels were overloaded and the presence of unspecific band on the height of oxidized form makes interpretation more difficult.

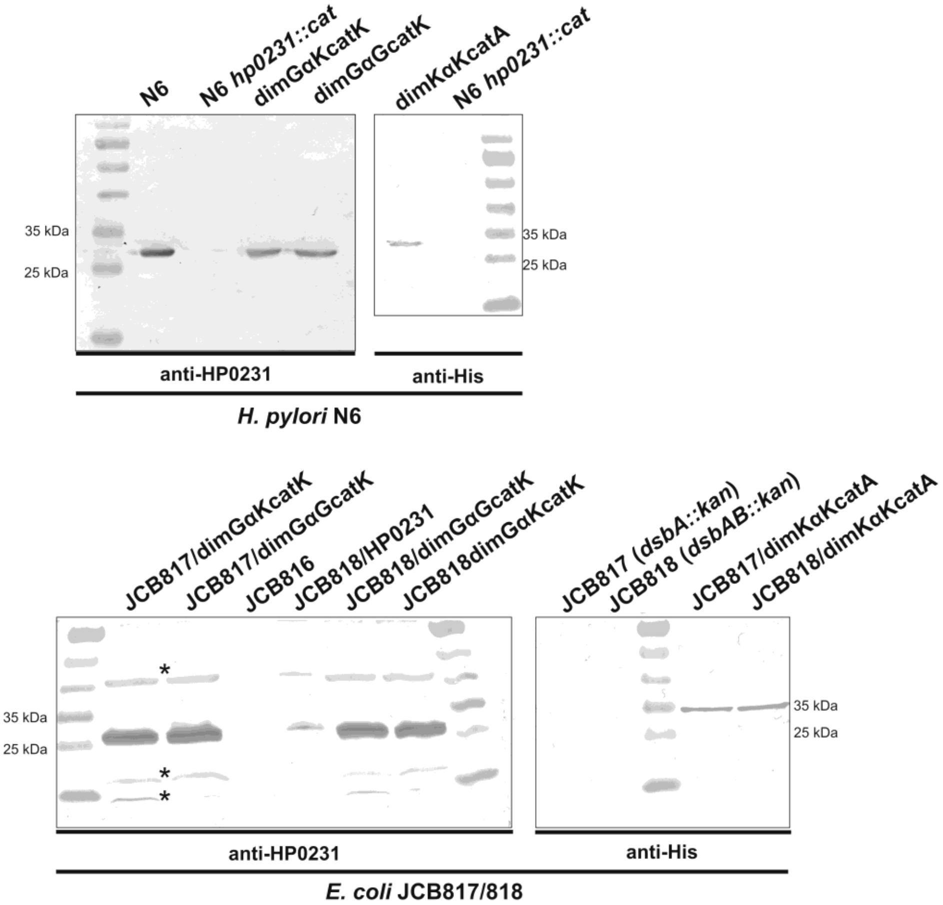

Figure S5.
Western-blot analysis of hybrid proteins in *H. pylori hp0231::cat* and *E. coli* JCB817/818 cells. Samples were separated by 12% SDS-PAGE, followed by Western-blot analysis using specific rabbit antibodies against HP0231 (chimeras dimGαKcatK and dimGαGcatK) or anti-His antibodies (chimera dimKαKcatA). Asterisk (*) mark unspecific bands.
